# Nursing actions indicative of autonomy and authority compatible with Advanced Nursing Practice in Brazil*

**DOI:** 10.1590/1518-8345.8245.4955

**Published:** 2026-10-07

**Authors:** Emerson Willian Santos de Almeida, Simone de Godoy, Maria Alice Barbosa Fortunato, Elaine Maria Leite Rangel Andrade, Ítalo Rodolfo Silva, Patrícia Sofia Oliveira Fonseca Nunes, Isabel Amélia Costa Mendes

**Affiliations:** 1 Universidade de São Paulo, Escola de Enfermagem de Ribeirão Preto, PAHO/WHO Collaborating Centre for Nursing Research Development, Ribeirão Preto, SP, Brazil.; 2 Organização Mundial da Saúde - África, WHO - AFRO, Guinea-Bissau.; 3 Universidade Federal do Piauí, Ininga, Teresina, PI, Brazil.; 4 Universidade Federal do Rio de Janeiro, Rio de Janeiro, RJ, Brazil.; 5 Universidade dos Açores, Escola Superior de Saúde, Angra do Heroísmo, Açores, Portugal.

**Keywords:** Primary Health Care, Professional Autonomy, Nursing, Ecological Study, Advanced Nursing Practice, Health Policies

## Abstract

**Objective:**

this study analyzed representative actions indicating autonomy and clinical authority among nurses in primary health care, compatible with Advanced Nursing Practice in Brazil.

**Method:**

the ecological study used secondary data from 2023 from the Brazilian Ministry of Health’s information system, covering all 26 states and the Federal District. Indicators related to consultations, medication prescriptions, diagnostic tests, and clinical referrals were examined.

**Results:**

the results reveal a wide range of activities performed by nurses in areas characteristic of Advanced Nursing Practice, while also showing significant geographical inequality, predominantly in the Southeast and South regions.

**Conclusion:**

the analysis reveals the effective exercise of advanced competencies, even without specific regulations, and highlights the need for public policies that promote specialized training, regulatory standardization, and the legal recognition of Advanced Nursing Practice to improve the quality and equity of health care.

## Highlights

**(1)** The results show that Advanced Nursing Practice is already being implemented in routine health services.

**(2)** 52 million nursing consultations; 37,000 medication prescriptions.

**(3)** 9.5 million diagnostic tests prescribed and 83 million clinical referrals.

**(4)** Effective performance of advanced practices, even without specific regulations.

**(5)** Fertile ground for establishing Professional Master’s programs in Advanced Nursing Practice, aligned with SUS and ICN.

## Introduction

The reconfiguration of the health workforce has intensified amid service overload, insufficient resources, a shortage of professionals, and increased demand driven by an aging population, the growth of chronic diseases, and recent pandemics. Progress toward Universal Health Coverage requires not only the strengthening of health systems, but also the mobilization of a skilled workforce capable of responding to the complex needs of the population^1^ ^)-(^ ^2^.

Nurses have been playing an increasingly strategic role, especially at the primary levels of health care, where comprehensive and longitudinal care is essential^3^.

The strengthening of the profession through investment in training, regulation, and the expansion of the scope of practice has been identified by international organizations as essential to achieving the Sustainable Development Goals (SDGs) and health equity. The World Health Organization (WHO) report State of the World’s Nursing 2025 highlights the strategic importance of nursing in achieving universal health coverage and recommends, among other measures, strengthening professional training and adopting Advanced Nursing Practice (ANP) to respond to the growing demands of health systems^1^. In this regard, evidence shows that advanced practice nurses can achieve clinical outcomes comparable to those of physicians, with high levels of patient satisfaction, especially in primary health care (PHC)^4^.

Although ANP originated in the United States in the 1960s, the concept developed and gained relevance in countries such as Canada, the United Kingdom, and Australia in the late 20th century, and has more recently spread to low- and middle-income countries. Its international adoption has been gradual and uneven across countries and regions, marked by persistent challenges such as role ambiguity, legal barriers, and institutional resistance^5^ ^)-(^ ^6^.

Internationally, reviews indicate that the transition of nurses to advanced roles in PHC is influenced by factors such as clarity of scope, stakeholder support, and the quality of interprofessional relationships^7^ ^)-(^ ^8^. Furthermore, considered a promising model for expanding access to and improving the effectiveness of health services, initially conceived for areas with a shortage of physicians and high demand for ongoing care, the authority to prescribe medications occupies a central place, as it represents both an expansion of the clinical scope and a symbolic milestone of professional recognition^8^, among other competencies that characterize the work of advanced practice nurses.

The International Council of Nurses (ICN)^8^ defines ANP as an expanded level of practice characterized by advanced clinical skills, more complex diagnostic judgment, autonomous decision-making, and authority to prescribe, order tests, and make referrals^8^ ^)-(^ ^9^. These competencies are based on specific training and regulatory requirements, and the ICN recommends that advanced practice nurses have at least a master’s degree in formally recognized programs, with curricula that include core subjects such as advanced pathophysiology, pharmacology, advanced physical examination, and diagnostic reasoning, in addition to a robust clinical workload focused on the management of complex conditions and independent decision-making^8^ ^)-(^ ^11^.

Although there is no specific regulation for ANP in Brazil, the National Primary Care Policy (PNAB, as per its acronym in Portuguese)^12^ assigns nurses activities that directly relate to these international domains, such as nursing consultations, diagnostic assessments, requesting tests, prescribing medications according to protocols, and referrals. Associated with the legal framework governing professional practice and regulations issued by the professional council^13^, these duties constitute an expanded clinical scope, which has concrete intersections with the competencies described by the ICN^8^, even though it lacks formal certification in advanced practice and a specific regulatory framework for this professional category^14^.

Actions representing nurses’ autonomy and authority, compatible with the practice of ANP, have been little explored in the Brazilian literature^14^. Considering the potential of ANP to strengthen health systems and the growing demand from nurses for greater clinical autonomy, it is essential to analyze how this practice has manifested in specific contexts, such as Brazil. Thus, autonomy is discussed in light of the legal and ethical foundations that should guide the scope of advanced nursing practices in Brazil. The authority of nurses, on the other hand, is related to the set of competencies involved in ANP, especially with regard to the range of knowledge and skills guided by the best scientific evidence, duly contextualized in time and space, that is, according to the profile of patients, available technologies, and professional expertise. Therefore, clinical authority refers to the prerogative to prescribe, request tests, and make referrals, competencies historically recognized as advanced domains in international models of advanced practice and exercised in Brazil under professional protocols and regulatory standards.

Thus, in this study, clinical autonomy is understood as the nurse’s ability to conduct consultations, perform diagnostic assessments, and make decisions in the care process, as provided for in national guidelines. Therefore, investigating key aspects, such as diagnostic authority and prescriptive responsibilities in the context of PHC, can help identify which clinical competencies nurses most frequently exercise, even in the absence of specific regulations for ANP. These data may be fundamental to support, among other developments, the updating of the National Curriculum Guidelines for undergraduate nursing programs, as well as to improve graduate programs and align professional training with the real demands of PHC. In addition, this analysis can strengthen the evidence base necessary for the consolidation of ANP in Brazil, promoting greater recognition, appreciation, and autonomy for nursing.

This study aimed to analyze representative actions of autonomy and clinical authority of nurses in primary health care, compatible with Advanced Nursing Practice in Brazil.

## Method

### Study design

This is an ecological study^15^, with an aggregated analysis unit comprising the Brazilian territory and its subdivision into 26 states and the Federal District of Brazil, in 2023.

### Secondary data sources and variables

Secondary public databases were used, extracted from the following national platforms: the Health Information System for Primary Care (SISAB, as per its acronym in Portuguese)^16^ and the National Registry of Health Facilities (CNES/Professionals)^17^. 

From these two databases, four indicators (14, 15, 16, and 17) related to ANP competencies were classified, and the selection, extraction, and organization of data followed a previously structured conceptual matrix derived from a previous study^18^, which establishes a relationship between the competencies of ANP, as proposed by the ICN^8^ and the PNAB^12^. The conceptual framework used in the study allowed establishing a correspondence between internationally recognized clinical competencies and the duties assigned to nurses by the law governing professional practice and by the PNAB^12^, which, through municipal protocols and regulations, authorize the conduct of consultations, the request of diagnostic tests, the prescription of medications, and referrals. 

For analytical purposes, concise operational definitions were adopted: clinical autonomy as the nurse’s ability to conduct consultations and perform diagnostic assessments; and clinical authority as the prerogative to prescribe, request tests, and refer users as per current regulatory frameworks. Based on these definitions, the indicators were classified according to their clinical nature: indicator 14 (consultations), representing clinical autonomy in the different areas covered by SISAB^16^, such as prenatal care, childcare, postpartum care, sexual and reproductive health, mental health, smoking, use of alcohol and other drugs, monitoring of communicable diseases, and screening activities; indicator 15 (test request), expressing diagnostic authority; indicator 16 (medication prescription), reflecting therapeutic authority; and indicator 17 (referrals), indicating expanded clinical authority in directing users to other points in the health care network. The four indicators represent advanced clinical dimensions, as detailed in Figure 1.

**Figure 1:** Filters used for data extraction in the Health Information System for Primary Care-2023

Geographic unit	Brazil
Period	January to December 2023
Report column	Indicator 14 = type of production: individual care; problem/condition assessed: prenatal care, childcare, postpartum care (up to 42 days), sexual and reproductive health, smoking, alcohol use, other drug use, mental health, communicable diseases: dengue fever; STDs; leprosy, tuberculosis; breast cancer screening, cervical cancer screening, cardiovascular risk screening
	Indicator 15 = type of production: individual care; problem/condition assessed: same as indicator 14; ICPC/ICD: ICPC (50) medication/prescription/renewal/injection, ICD (Z760) repeat prescription and issuance
	Indicator 16 = type of production: procedure; procedure: test. proteinuria rapid test, HIV rapid test, pregnancy rapid test, hepatitis C rapid test, syphilis rapid test, and ophthalmologic screening
	Indicator 17 = type of production: individual care; problem/condition assessed: same as indicator 14; conduct: scheduling for NASF*, scheduling for groups, discharge from episode, internal referral on the same day, intersectoral referral, referral to CAPS, referral for hospital admission, referral for home care service, referral for specialized care, referral for emergency care, return for continued/scheduled care, return for scheduled consultation
Type of care	Scheduled appointment. Prog/cont. care, scheduled appointment, initial/orientation request, same-day appointment request, urgent care request
Professional category	Nurse
Care sector	BHU, Mobile unit, Street, Home, School/daycare center, Others, Health center, Institution/shelter, Prison or similar facility, Socio-educational facility
Type of healthcare team	Family Health Team (ESF), Community Health Agent Team (EACS), NASF*/eMulti, Primary Care Team (EAB), Street Clinic Team (ECR), Oral Health Team (SB), Prison Primary Care Team (EABp), Primary Care Team - eAP

*NASF = Family Health Support Center, as per its acronym in Portuguese

It is recognized that these indicators correspond to administrative production records rather than details on the clinical complexity of each action. Nevertheless, they represent the structural dimensions of the legal and regulatory scope of nursing practice in PHC, allowing for the analysis of the territorial distribution of these activities and their alignment with competencies described in international references. 

### Collection procedures

The extraction of SISAB data was carried out between November 3 and 5, 2024, through an automated, parameterized process that applied a single standardized filter across all states, team modalities, and care units, according to the system’s structural options. Records for the 12 months of 2023 were selected, restricted to professionals in the nursing category and covering the different modalities authorized in PHC [Family Health Team (ESF), Primary Care Team (eAP), Street Clinic Team (eCR), and other recognized teams], according to the standardized codes and parameters of the Management System for the Table of Procedures, Medications, and Orthoses, Prostheses, and Special Materials (SIGTAP) of the Brazilian Unified Health System (SUS), as described in Figure 1. This procedure ensured uniformity in selection and reduced heterogeneity caused by coding differences across regions and team types.

After extraction, the database underwent double validation, conducted by a statistician and a second researcher responsible for checking and manipulating the database, ensuring the integrity, consistency, and applicability of the records. As the data were aggregated by type of care, date, and procedure, according to the official SISAB parameters, no duplicates were found within the established criteria. Incomplete, invalid records or those incompatible with PHC were automatically excluded by the filter applied during extraction.

In addition, geospatial data (maps and geographic coordinates) were obtained from the website of the Brazilian Institute of Geography and Statistics (IBGE, as per its acronym in Portuguese)^19^, using geographic projection. Information related to the general population size for each state and the total Brazilian population (census year) was compiled. Digital maps and their respective geodetic coordinates (files in *.shp, *.shx, and *.prj formats) of the national territory and its subdivision into states (reference year 2017) were obtained from http:downloads.ibge.gov.br/downloads_geociencias.htm. Files with the .shp extension assume the SIRGAS_2000 geographic projection, *datum*[“d_SIRGAS_2000”, *spheroid*[“grs_1980”,6378137,298.257222101], *primem*[“*Greenwich*”,0], *unit*[“*degree*”, 0.017453292519943295]].

### Data analysis

This stage was divided into two phases.

#### Phase 1 - Descriptive analysis

The absolute frequencies of activities associated with the selected indicators were quantified by state. This phase allowed us to estimate the volume of actions performed by nurses in the PHC setting, classified by advanced practice domain (Figure 2). We also used the nursing workforce linked to PHC registered in the National Register of Health Facilities - CNES/Professionals^17^ ^)^ as a reference. The extraction was performed using data available for July 2023, totaling 100,614 nurses working in PHC across the country. 

**Figure 2:** Absolute frequency of nurses and interventions, in terms of consultation, management of complex care episodes, authority to diagnose, prescribe medications, diagnostic tests, and therapeutic treatments, in the 26 states of the country and the Federal District. Brazil, 2023

FU	State	Number of Nurses	Indicator 14*	Indicator 15^†^	Indicator 16^‡^	Indicator 17^§^
1	Acre	477	304027	4	128971	371863
2	Alagoas	1812	1035600	213	251660	1904659
3	Amapá	556	93576	178	31724	189465
4	Amazonas	1627	1346253	259	452464	2059683
5	Bahia	6604	3873064	1285	583719	6076458
6	Ceará	4582	2963851	1190	450512	5990449
7	Federal District	1618	601603	45	88536	887839
8	Espírito Santo	1861	983349	4666	131563	1586019
9	Goiás	2985	1481209	3664	220240	2538890
10	Maranhão	3686	1940515	650	518498	3427834
11	Mato Grosso	1673	1012717	153	226878	1606304
12	Mato Grosso do Sul	1326	722841	40	178672	1113670
13	Minas Gerais	11927	4975276	3876	771176	9215929
14	Pará	3456	1753622	442	378462	2772583
15	Paraíba	2474	1244622	601	121172	2229152
16	Paraná	6431	3405789	2409	591524	4931203
17	Pernambuco	4148	2983259	165	366765	4587135
18	Piauí	2361	950709	17	48363	1906376
19	Rio de Janeiro	5726	3858600	5765	653215	5390978
20	Rio Grande do Norte	2033	1023808	172	147694	1574712
21	Rio Grande do Sul	6707	3578698	3161	916419	5090050
22	Rondônia	794	372872	46	139102	480213
23	Roraima	381	105395	0	58952	160319
24	Santa Catarina	4842	2541553	1260	735644	3936650
25	São Paulo	18216	7913901	6523	1141077	11242152
26	Sergipe	1236	640694	264	120886	947929
27	Tocantins	1075	421043	164	58906	807368
	General Total	100.614	52.128.446	37.212	9.512.794	83.025.882

*Indicator 14 = Nursing Consultation; ^†^Indicator 15 = Medication prescriptions; ^‡^Indicator 16 = Test requests; ^§^Indicator 17 = Referrals

#### Phase 2 - Calculation of indicators and georeferencing

Based on the total information (Figure 2), two indices were constructed:

1) Adjusted proportion: calculated via multiple linear regression, adjusted for the size of the nurse population. This technique was used to estimate the expected number of actions for each indicator evaluated, accounting for the size of the nurse population (covariate) in each state, to minimize the confounding effect of the uneven distribution of these professionals across the country. The index was calculated according to the equation:

$$Adjusted\;proportion=\frac{expected\;N\;of\;the\;Indicator\;in\;Figure\;2}{overall\;Indicator\;score}*100$$

The goodness-of-fit of the linear regression models was assessed using R^2^ and adjusted R^2^ (both above 90% in the four models), and the homoscedasticity of the residuals (data not shown). This index allows comparison of the indicators in Figure 2 within states.

2) Standardized rate: estimated by direct standardization^15^, using the total Brazilian population (census year) as the standard population. Based on the totals shown in Figure 2, the crude proportions [(observed N of the indicator/total indicator)] were calculated, and the indices were estimated according to the equation:

$$Standardized\;rate=\frac{crude\;proportion\;of\;the\;Indicator}{standard\;population}*10^{n}\;inhabitants/year$$

This index allows for comparison in two directions-within and between-federal units. For plotting on thematic maps^20^, this index was classified into quarters, according to the quartiles of the distribution. 

All analyses were performed using Stata^®^ software, version 13.2, with the spmap command package^21^ for the georeferencing stage of standardized rates. 

### Ethical considerations

As this study is based exclusively on secondary, aggregated data in the public domain, approval by an ethics committee was not required, in accordance with the exemption provided for in CNS Resolution No. 510/2016.

## Results

Table 1 presents the adjusted proportional distribution of the main actions performed by nurses in PHC, considering the exercise of competencies consistent with ANP. In all states, the indicator that stood out most in magnitude was 16, i.e., authority to prescribe diagnostic tests and therapeutic treatments, with the exception of the states of Rio de Janeiro and São Paulo.

**Table 1:** Adjusted proportions of indicators, by state. Brazil, 2023

State	Adjusted Proportions (%)*			
	Indicator 14	Indicator 15	Indicator 16	Indicator 17
Acre	0.7	0.3	1.0	0.8
Amapá	0.8	0.4	1.1	0.8
Amazonas	2.4	2.0	2.6	2.4
Pará	3.0	2.7	3.2	3.0
Rondônia	1.1	0.8	1.4	1.2
Roraima	0.7	0.3	1.0	0.7
Tocantins	1.4	1.0	1.6	1.4
Alagoas	1.8	1.4	2.0	1.8
Bahia	6.6	6.5	6.7	6.6
Ceará	4.5	4.2	4.6	4.5
Maranhão	3.0	2.7	3.1	2.9
Paraíba	2.7	2.3	2.9	2.7
Pernambuco	5.1	4.9	5.3	5.1
Piauí	1.9	1.6	2.2	1.9
Rio Grande do Norte	1.9	1.6	2.2	1.9
Sergipe	1.5	1.2	1.8	1.6
Espírito Santo	2.0	1.7	2.3	2.0
Minas Gerais	8.8	8.7	8.8	8.7
Rio de Janeiro	9.1	9.1	9.1	9.1
São Paulo	24.0	24.7	23.5	24.0
Paraná	5.0	4.8	5.1	5.0
Rio Grande do Sul	4.6	4.4	4.8	4.6
Santa Catarina	3.2	2.9	3.4	3.2
Federal District	3.1	2.8	3.3	3.1
Goiás	3.2	2.9	3.4	3.2
Mato Grosso	2.0	1.7	2.3	2.0
Mato Grosso do Sul	1.6	1.3	1.9	1.7

*Adjustment for the size of the nurse population

Table 2 shows the standardized rates of the indicators by state. Interregional comparisons reveal that São Paulo leads in all indicators (ranging from 5.6 to 8.2), followed by Minas Gerais, Rio de Janeiro, and Paraná. 

**Table 2:** Distribution of standardized rates by state. Brazil, 2023

State	Standardized Rates*			
	Indicator 14	Indicator 15	Indicator 16	Indicator 17
Acre	0.3	0	0.6	0.2
Amapá	0.1	0.2	0.2	0.1
Amazonas	1.2	0.3	2.2	1.2
Pará	1.6	0.6	1.9	1.6
Rondônia	0.3	0.1	0.7	0.3
Roraima	0	0	0.3	0.1
Tocantins	0.4	0.2	0.3	0.5
Alagoas	0.9	0.3	1.2	1.1
Bahia	3.5	1.6	2.9	3.4
Ceará	2.7	1.5	2.2	3.4
Maranhão	1.7	0.8	2.6	1.9
Paraíba	1.1	0.7	0.6	1.3
Pernambuco	2.7	0.2	1.8	2.6
Piauí	0.8	0	0.2	1.1
Rio Grande do Norte	0.9	0.2	0.7	0.9
Sergipe	0.6	0.3	0.6	0.5
Espírito Santo	0.9	5.9	0.6	0.9
Minas Gerais	4.5	4.9	3.8	5.2
Rio de Janeiro	3.5	7.3	3.2	3.0
São Paulo	7.1	8.2	5.6	6.4
Paraná	3.1	3.0	2.9	2.8
Rio Grande do Sul	3.2	4.0	4.5	2.9
Santa Catarina	2.3	1.6	3.6	2.2
Federal District	0.5	0.1	0.4	0.5
Goiás	1.3	4.6	1.1	1.4
Mato Grosso	0.9	0.2	1.1	0.9
Mato Grosso do Sul	0.6	0.1	0.9	0.6

*Standardization by the direct method, standard population: total Brazilian population

Figure 3 graphically presents the spatial distribution of standardized rates in quartiles, allowing for the geographical visualization of ANP-compatible actions in PHC. A pattern of higher concentration of indicators is observed in the states that make up the Southeast and South regions, with emphasis on São Paulo, Minas Gerais, Rio de Janeiro, Paraná, Rio Grande do Sul, and Santa Catarina. In contrast, the North Region and parts of the Northeast show lower concentrations of ANP performance indicators. The visualization shows a geographic gradient of inequality in the exercise of nurses’ autonomy and clinical authority. This pattern suggests contexts that are more favorable to the consolidation of ANP, and others that are less favorable, where clinical practices need to be reviewed and expanded, provided they are supported by municipal regulatory protocols and structural investments in specialized training and continuing professional development. 

**Figure 3:**
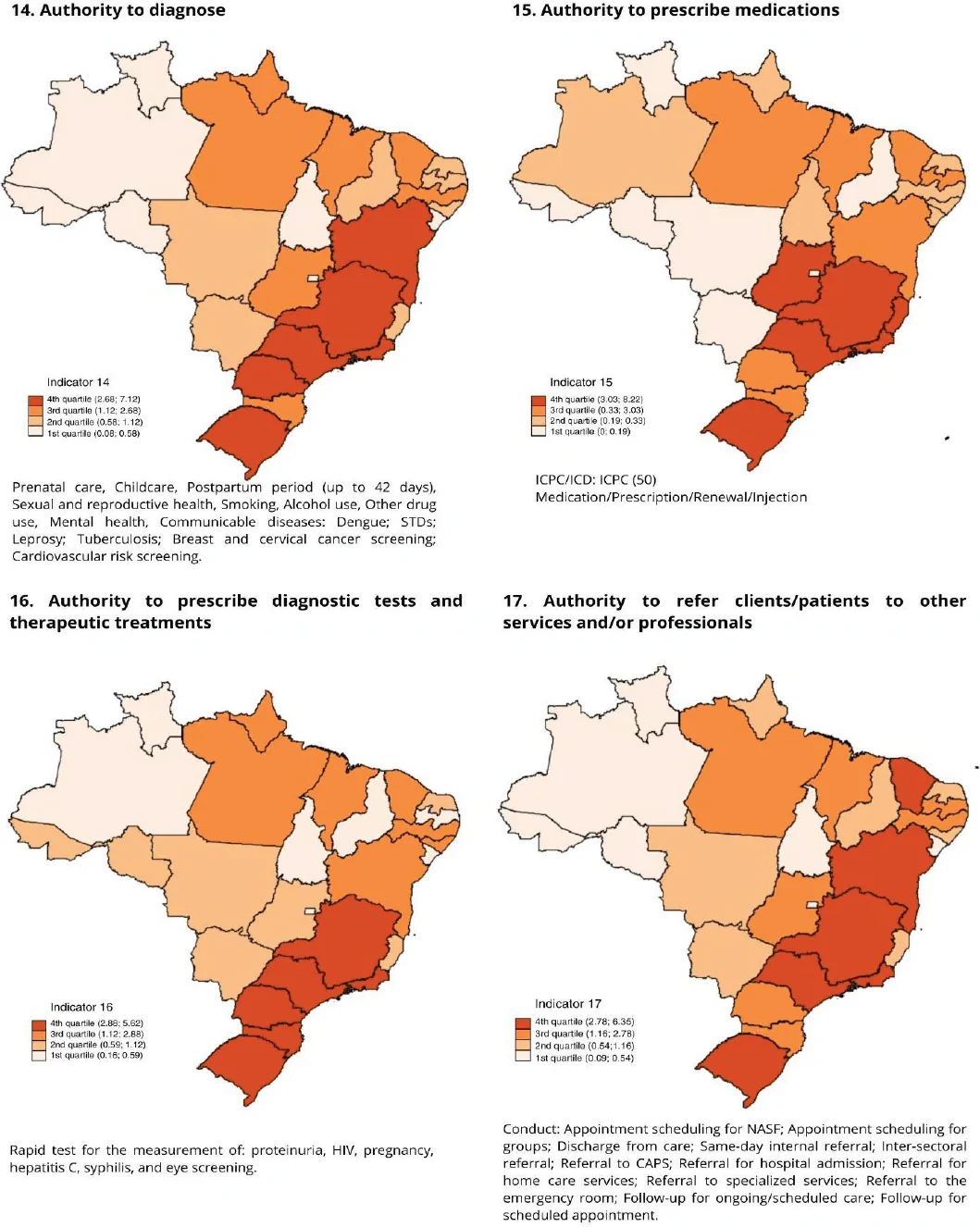
Geographic distribution, by state, of indicators for consultation, management of complex care episodes, authority to diagnose, prescribe medications, diagnostic tests, and therapeutic treatments performed by nurses in primary health care. Brazil, 2023

## Discussion

The actions performed by nurses in PHC are aligned with the guidelines established by the ICN^8^ for advanced nursing practice. This study focused on analyzing actions that reflect nurses’ clinical authority and autonomy: conducting consultations, clinical referrals, requesting diagnostic tests, and prescribing medications in the context of PHC.

The distribution of these four sets of actions, in terms of volume of records, occurred in the following order: nursing consultation, referral of users to other health professionals and/or services, request of diagnostic tests, and, finally, prescription of medications. In an ecological study, inferences refer to collective units rather than the individual performance of professionals. The possibility of biases related to the quality of records and the structural heterogeneity of services must be considered, despite the adoption of standardization measures.

Figure 3 is central to understanding the geographic pattern of implementation of the four types of action aligned with ANP in Brazil. The image shows a concentration of the four indicators in the South-Southeast axis, suggesting greater institutionalization of advanced practices in these territories. On the other hand, states in the North and Northeast regions, such as Acre, Roraima, Piauí, and Amapá, are in the lower quartiles, indicating a lower density of clinical actions compatible with ANP, which may reflect structural limitations, a scarcity of local protocols, or underreporting. This gradient shows that, although the actions developed by nurses in PHC in Brazil align with the assumptions of ANP, significant regional inequalities persist across states. Thus, the expansion of ANP seems to depend on investments in professional training, local regulation, and public financing.

The significant number of nursing consultations recorded (52,128,446) demonstrated the wide range of clinical activities performed by nurses, especially in conditions that require greater diagnostic autonomy, such as women’s health, mental health, communicable diseases, and cancer screening. Although this practice is already consolidated in some territories, it is unevenly distributed among the states, with São Paulo, Minas Gerais, Rio de Janeiro, Paraná, Bahia, and Rio Grande do Sul standing out, as shown in Figure 3 (upper quartile of the spatial analysis). These findings reinforce the existence of contexts more favorable to the consolidation of ANP in these states.

National studies corroborate this scenario by highlighting the positive perception of nurse autonomy in PHC^14^ ^),(^ ^22^ ^)-(^ ^24^, and showing that, when supported by regulations and scientific evidence, these professionals take on a leading role in clinical care, especially in sensitive areas such as sexually transmitted infections (STIs) and women’s health care^25^. 

Thus, the results presented in this study do not correspond to a formally recognized model of Advanced Nursing Practice in Brazil, given that this function lacks specific regulation, but they highlight dimensions of nurses’ clinical practice that align with competencies described in international frameworks and point to potential for development. These actions, performed by generalist nurses within the expanded scope permitted by Brazilian legislation, reveal patterns of clinical maturity and leadership that can support the development of training and regulatory frameworks aligned with international ANP guidelines. The results demonstrate a significant set of practices that engage with advanced domains, providing an empirical basis for guiding future debates and proposals.

In addition, 83,025,882 referral records were identified, highlighting the role of nurses as care coordinators and facilitators of care flows. Quartile analysis showed a higher concentration of these records in the states of Ceará, Bahia, Minas Gerais, São Paulo, Rio de Janeiro, and Rio Grande do Sul, as shown in Figure 3. This concentration may be related to the greater institutionalization of municipal clinical protocols that support nurses’ clinical decisions. The literature^26^ corroborates this finding by highlighting the role of the “liaison nurse” as a key element in the effective coordination of healthcare networks, especially in referral and counter-referral processes.

Regarding the prescription of diagnostic tests and therapeutic treatments (n=9,512,794), the results indicated greater expression in the states of Minas Gerais, Rio de Janeiro, São Paulo, Paraná, Santa Catarina, and Rio Grande do Sul, suggesting better structural and organizational conditions, such as greater coverage of the Family Health Strategy (ESF, as per its acronym in Portuguese) and technical training capacity. 

On the other hand, the results regarding the prescription of medications by nurses (n=37,212) were relatively low compared to the volume of consultations and referrals. Although the PNAB authorizes nurses to prescribe, the implementation of this competency depends on local protocols, leading to disparities between states. Figure 3 shows a higher incidence of prescriptions in the states of São Paulo, Rio de Janeiro, Espírito Santo, Minas Gerais, Goiás, and Paraná. Previous studies^9^ ^),(^ ^14^ ^),(^ ^27^ indicate that nurses feel prepared and recognize prescribing as an advance in professional autonomy, provided it is supported by technical regulations. Therefore, the low national figures for nurse prescribing may be related to the absence or outdated nature of municipal protocols, legal uncertainty, and institutional barriers. It should be noted that prescribing medications in the context of Advanced Nursing Practice in PHC represents an advance in line with the principles of comprehensive and effective care^9^ ^),(^ ^14^. 

In this context, the ICN recommends the inclusion of drug prescription within the scope of action of ANP, at a global level, provided that it is accompanied by specific training and regulation, as well as institutional support^8^. 

In Europe, the implementation of ANP varies greatly, with only 11 of the 35 countries in the pan-European region having formal legislation on the subject^28^. There is still considerable variability in the definition of ANP, especially regarding nomenclature, scope of action, regulation, educational requirements for exercising the function, and financing strategies^28^, which compromises its implementation. In countries such as France, for example, the ambiguity of roles, combined with uncertainty regarding the financing model and institutional support, hinders the development of ANP, a scenario also seen in countries such as England, where the practice is widely established^29^ ^)-(^ ^30^. In Nordic and Oceanic countries, such as Australia, Finland, and Norway, there is greater standardization in the training, regulation, and recognition of ANP as strategic for health systems^31^. A European study highlights the importance of combating professional isolation through networking and mentoring as essential strategies for promoting the sustainability of the advanced practice nursing workforce^32^. In the region of the Americas, 64% of countries have implemented advanced nursing functions^33^. However, Latin American countries should increase investment in this strategy^14^ ^),(^ ^34^, valuing dialogue between stakeholders^35^.

In contexts where discussion about ANP is still incipient, although they consider it a promising initiative^36^ ^)-(^ ^37^, it is essential to strengthen the educational, political, and institutional foundations of nursing to ensure the sustainable and effective introduction of advanced practice^38^. Additionally, continuing education was considered a key factor in fostering confidence in the performance of advanced practice nursing, as demonstrated in studies that analyzed the type of educational intervention over the course of implementation^39^. In the Brazilian case, it is precisely the educational base that should become the focus of strategic actions by nursing leaders, since, even though there is a growing number of advanced clinical interventions in PHC, the absence of specific national regulations, coupled with dependence on local regulations, compromises equity in the practice of ANP among states. The study also pointed out that there is a critical mass in place to move forward: with more than 100,000 nurses working in PHC, which should be associated with a robust nursing education system (undergraduate, master’s, and doctoral academic and professional courses)^40^ and leaders organized into specialties and scientific societies.

Despite its national scope and the robustness of the data used, this study has some limitations that should be considered. The main one concerns possible underreporting of actions recorded in official health information systems, especially in the North and Northeast regions, as observed in the states of Piauí, Roraima, and Acre, where the standardized indicators were zero or extremely low. This scenario may not reflect the absence of clinical practices compatible with ANP, but rather weaknesses in recording, inconsistencies in information systems, or the absence of standardized coding in health units. In addition, the ecological and aggregate nature of the analysis prevents causal inferences or individualized interpretations. Thus, additional studies are needed to provide disaggregated information.

It is also important to note that the actions analyzed are not, in themselves, equivalent to Advanced Nursing Practice. In the Brazilian context, many of them are part of the expanded scope of generalist nurses in the SUS, as provided for in the PNAB and the law on professional practice, and do not necessarily imply advanced competencies as defined by the ICN. 

Nevertheless, the consistent and widespread implementation of the actions analyzed in this study highlights the clinical maturity that nurses have been developing in PHC, demonstrating technical capacity, accountability, and leadership in care. These findings also reveal significant potential for guiding policies on training, regulation, and the expansion of the professional scope by offering concrete data on nursing performance in Brazil. Thus, the indicators analyzed clearly support the central role of nurses in Brazilian PHC and contribute to strategic debates on the regulation and strengthening of advanced practice in the country, without, however, directly characterizing such actions as ANP, but highlighting clinical dimensions that dialogue with this international framework.

## Conclusion

This ecological study provides a national overview of the distribution and intensity of nursing clinical actions in primary health care. Although it does not allow for inferring the complexity of clinical reasoning or for characterizing advanced competencies as defined by the ICN, the patterns observed reveal a robust field of practice in which nursing plays a central role in care and accumulates experience that can support structural advances.

The identified territorial heterogeneity indicates the influence of organizational capacities, infrastructure, and the availability of professionals, highlighting areas that require strengthening and capacity building. In addition, the presence of more than 100,000 nurses working in PHC, associated with a solid educational structure and a consolidated critical mass at the academic and professional postgraduate levels, constitutes a strategic scenario for the development of advanced training programs, including Professional Master’s programs oriented toward expanded clinical practice and aligned with the needs of the SUS.

The findings point to concrete ways to improve nursing clinical practice in Brazil, including improving health information recording systems, standardizing coding, harmonizing protocols, and conducting qualitative and mixed-methods studies to deepen understanding of the decision-making processes and conditions that shape professional autonomy in PHC. By offering empirical evidence on the clinical work of nurses on a national scale, this study contributes to strategic debates on strengthening and expanding the clinical scope, reinforcing the relevance of measures that advance toward the regulation and recognition of nursing autonomy and clinical authority, which are central elements for the effectiveness and sustainability of primary health care in Brazil.

## Data Availability

All data generated or analysed during this study are included in this published article.
