# Supplementary material for: Nursing actions indicative of autonomy and authority compatible with Advanced Nursing Practice in Brazil
Source: Rev Lat Am Enfermagem. 2026 Oct 7;34:e4955. doi: 10.1590/1518-8345.8245.4955 (PMC13679910; doi:10.1590/1518-8345.8245.4955)
Supplement: Translation

# Translation: Ações do enfermeiro indicativas de autonomia e autoridade compatíveis com Prática Avançada da Enfermagem brasileira*

**DOI:** 10.1590/1518-8345.8245.4956

**Authors:** Emerson Willian Santos de Almeida, Simone de Godoy, Maria Alice Barbosa Fortunato, Elaine Maria Leite Rangel Andrade, Ítalo Rodolfo Silva, Patrícia Sofia Oliveira Fonseca Nunes, Isabel Amélia Costa Mendes

**Affiliations:** 1 Universidade de São Paulo, Escola de Enfermagem de Ribeirão Preto, Centro Colaborador da OPAS/OMS para o Desenvolvimento da Pesquisa em Enfermagem, Ribeirão Preto, SP, Brasil.; 2 Organização Mundial da Saúde - África, WHO - AFRO, Guiné-Bissau.; 3 Universidade Federal do Piauí, Ininga, Teresina, PI, Brasil.; 4 Universidade Federal do Rio de Janeiro, Rio de Janeiro, RJ, Brasil.; 5 Universidade dos Açores, Escola Superior de Saúde, Angra do Heroísmo, Açores, Portugal.

**Keywords:** Atenção Primária à Saúde, Autonomia Profissional, Enfermagem, Estudo Ecológico, Prática Avançada de Enfermagem, Políticas de Saúde

## Abstract

**Objetivo:**

este estudo analisou ações representativas de autonomia e autoridade clínica de enfermeiros na Atenção Primária à Saúde compatíveis com a Prática Avançada de Enfermagem no Brasil.

**Método:**

a pesquisa ecológica utilizou dados secundários de 2023 provenientes do sistema de informação do Ministério da Saúde do Brasil, considerando os 26 estados e o Distrito Federal. Foram examinados indicadores de consultas, prescrições de medicamentos e testes diagnósticos, além de encaminhamentos clínicos.

**Resultados:**

os resultados revelam uma ampla atuação dos enfermeiros em domínios característicos de Prática Avançada de Enfermagem expressando, no entanto, desigualdade geográfica expressiva predominante nas regiões Sudeste e Sul.

**Conclusão:**

a análise revela o exercício efetivo de competências avançadas, mesmo sem regulamentação específica, e destaca a necessidade de políticas públicas que promovam formação especializada, padronização normativa e reconhecimento legal da Prática Avançada de Enfermagem, visando à qualificação e equidade dos cuidados em saúde.

## Destaques

**(1)** O resultado sustenta que a Prática Avançada da Enfermagem já se concretiza no cotidiano dos serviços.

**(2)** 52 milhões de consultas de Enfermagem; 37 mil prescrições de medicamentos.

**(3)** 9,5 milhões de testes diagnósticos prescritos e 83 milhões de encaminhamentos clínicos.

**(4)** Exercício efetivo de ações avançadas, mesmo sem regulamentação específica.

**(5)** Terreno fértil para instituir Mestrados Profissionais em Prática Avançada da Enfermagem, alinhados ao SUS e ao ICN.

## Introdução

A reconfiguração da força de trabalho em saúde tem se intensificado diante da sobrecarga dos serviços, insuficiência de recursos, escassez de profissionais e aumento da demanda em contexto de envelhecimento populacional, crescimento das doenças crônicas e pandemias recentes. O avanço rumo à Cobertura Universal de Saúde requer não apenas o fortalecimento dos sistemas de saúde, mas também a mobilização de força de trabalho qualificada, com capacidade para responder às complexas necessidades da população^1^ ^)-(^ ^2^.

Os enfermeiros vêm desempenhando um papel cada vez mais estratégico, sobretudo nos níveis primários de atenção à saúde, nos quais a integralidade e a longitudinalidade do cuidado são fundamentais^3^.

A valorização da profissão, por meio do investimento em formação, regulação e expansão do escopo de prática, tem sido apontada por organismos internacionais como eixo essencial para o alcance dos Objetivos do Desenvolvimento Sustentável (ODS) e da equidade em saúde. O relatório *State of the World’s Nursing 2025* da Organização Mundial da Saúde (OMS) destaca a importância estratégica da Enfermagem para o alcance da cobertura universal de saúde e recomenda, entre outras medidas, o fortalecimento da formação profissional e a adoção da Prática Avançada de Enfermagem (PAE) como resposta às crescentes demandas dos sistemas de saúde^1^. Nesse sentido, evidências demonstram que enfermeiros de prática avançada podem alcançar resultados clínicos comparáveis aos de médicos, com níveis elevados de satisfação entre os pacientes, especialmente na Atenção Primária à Saúde (APS)^4^.

Embora a PAE tenha origem nos Estados Unidos, nos anos 1960, o conceito foi se desenvolvendo e ganhando relevância em países como o Canadá, Reino Unido e Austrália, no final do século XX, tendo-se mais recentemente estendido a países de baixa e média renda. A sua adoção internacional vem ocorrendo de forma gradativa e desigual em cada país e região, marcada por desafios persistentes, como ambiguidade de papéis, entraves legais e resistência institucional^5^ ^)-(^ ^6^.

Internacionalmente, revisões indicam que a transição de enfermeiros para funções avançadas na APS é impactada por fatores, como clareza do escopo, apoio das partes interessadas e qualidade das relações interprofissionais^7^ ^)-(^ ^8^. Ademais, considerada um modelo promissor para a ampliação do acesso e da resolutividade dos serviços, inicialmente pensada a partir de áreas com escassez de médicos e alta demanda por cuidados continuados, a autoridade para prescrever medicamentos ocupa lugar central, por representar tanto ampliação do escopo clínico quanto marco simbólico de reconhecimento profissional^8^, entre outras competências que caracterizam a atuação de enfermeiros de prática avançada.

O *International Council of Nurses* (ICN)^8^ define a PAE como um nível ampliado de prática caracterizado por competências clínicas avançadas, julgamento diagnóstico mais complexo, tomada de decisão autônoma e autoridade para prescrever, solicitar exames e realizar encaminhamentos^8^ ^)-(^ ^9^. Essas competências se sustentam em requisitos específicos de formação e regulação, e o ICN recomenda que enfermeiros de prática avançada tenham, no mínimo, titulação em nível de mestrado, em programas formalmente reconhecidos, com currículos que incluam disciplinas centrais como fisiopatologia avançada, farmacologia, exame físico avançado e raciocínio diagnóstico, além de carga horária clínica robusta voltada ao manejo de condições complexas e à tomada de decisão independente^8^ ^)-(^ ^11^.

Embora não exista regulamentação específica para a PAE no Brasil, a Política Nacional de Atenção Básica (PNAB)^12^ atribui aos enfermeiros atividades que dialogam diretamente com esses domínios internacionais, tais como a consulta de enfermagem, a avaliação diagnóstica, a solicitação de exames, a prescrição de medicamentos em protocolos e o encaminhamento. Associadas ao respaldo da lei do exercício profissional e das normativas do conselho de classe^13^, essas atribuições configuram um escopo clínico ampliado que apresenta interseções concretas com as competências descritas pelo ICN^8^, ainda que desprovido de titulação formal em prática avançada e de um marco regulatório específico para essa categoria profissional^14^.

As ações representativas de autonomia e autoridade do enfermeiro, compatíveis com o exercício da PAE, têm sido pouco exploradas na literatura brasileira^14^. Considerando o potencial da PAE para fortalecer os sistemas de saúde e a crescente demanda dos enfermeiros por maior autonomia clínica, torna-se essencial analisar como essa prática vem se manifestando em contextos específicos, como o brasileiro. Desse modo, fala-se em autonomia a partir das bases legais e deontológicas que devam orientar o escopo das práticas avançadas do enfermeiro, no Brasil. Já a autoridade do enfermeiro está relacionada ao conjunto de competências que envolvem as PAEs, especialmente no que concerne ao rol de conhecimentos e habilidades orientados pelas melhores evidências científicas, devidamente contextualizadas no tempo e espaço, ou seja, de acordo com o perfil dos pacientes, tecnologias disponíveis e expertise do profissional; portanto, autoridade clínica refere-se à prerrogativa de prescrever, solicitar exames e realizar encaminhamentos, competências historicamente reconhecidas como domínios avançados em modelos internacionais de prática avançada e exercidas no Brasil de forma regulada por protocolos e normativas profissionais.

Assim, neste estudo, autonomia clínica é entendida como a capacidade do enfermeiro de conduzir consultas, realizar avaliação diagnóstica e tomar decisões no processo de cuidado, tal como previsto nas diretrizes nacionais. Portanto, investigar aspectos-chave, como a autoridade diagnóstica e as responsabilidades prescritivas no contexto da APS, pode contribuir para identificar quais competências clínicas os enfermeiros exercem com maior frequência, mesmo na ausência de uma regulamentação sobre PAE. Esses dados podem ser fundamentais para subsidiar, entre outros desdobramentos, a atualização das Diretrizes Curriculares Nacionais para a graduação em Enfermagem, além de aprimorar os programas de pós-graduação e alinhar a formação profissional às demandas reais da APS. Além disso, essa análise pode fortalecer a base de evidências necessária para a consolidação da PAE no Brasil, promovendo maior reconhecimento, valorização e autonomia para a enfermagem.

Este estudo teve como objetivo analisar ações representativas de autonomia e autoridade clínica de enfermeiros na Atenção Primária à Saúde compatíveis com a Prática Avançada de Enfermagem no Brasil.

## Método

### Desenho do estudo

Trata-se de um estudo ecológico^15^, com unidade de análise agregada, compreendendo o território brasileiro e sua subdivisão em 26 estados e o Distrito Federal do Brasil, no ano de 2023.

### Fontes secundárias de dados e variáveis

Foram utilizadas bases públicas secundárias extraídas das seguintes plataformas nacionais: Sistema de Informação em Saúde para a Atenção Básica (SISAB)^16^ e Cadastro Nacional de Estabelecimentos de Saúde (CNES/Profissionais)^17^. 

A partir dessas duas bases de dados, foram classificados quatro indicadores (14, 15, 16 e 17) relacionados com as competências da PAE, e a seleção, extração e organização dos dados seguiram uma matriz conceitual previamente estruturada, derivada de estudo anterior^18^, a qual estabelece relação entre as competências da PAE, conforme proposto pelo ICN^8^ e os dispositivos normativos da PNAB^12^ brasileira. A matriz conceitual utilizada no estudo permitiu estabelecer a correspondência entre competências clínicas reconhecidas internacionalmente e as atribuições conferidas aos enfermeiros pela lei do exercício profissional e pela PNAB^12^, que, mediante protocolos e regulamentações municipais, autorizam a condução de consultas, solicitação de exames, prescrição de medicamentos e realização de encaminhamentos. 

Para fins analíticos, adotaram-se definições operacionais sintéticas: autonomia clínica como a capacidade do enfermeiro de conduzir consultas e realizar avaliação diagnóstica; e autoridade clínica como a prerrogativa de prescrever, solicitar exames e encaminhar usuários conforme marcos normativos vigentes. Com base nessas definições, os indicadores foram classificados segundo sua natureza clínica: indicador 14 (consultas), representando autonomia clínica nas diferentes áreas previstas no SISAB^16^, como pré-natal, puericultura, puerpério, saúde sexual e reprodutiva, saúde mental, tabagismo, uso de álcool e outras drogas, acompanhamento de doenças transmissíveis e ações de rastreamento; indicador 15 (solicitação de exames), expressando autoridade diagnóstica; indicador 16 (prescrição de medicamentos), refletindo autoridade terapêutica; e indicador 17 (encaminhamentos), indicando autoridade clínica ampliada no direcionamento do usuário a outros pontos da rede assistencial. Os quatro indicadores representam dimensões clínicas avançadas, conforme detalhado na Figura 1.

**Figura 1:** Filtros utilizados para extração de dados no Sistema de Informação em Saúde para a Atenção Básica-2023

Unidade geográfica	Brasil
Período	Janeiro a dezembro de 2023
Coluna no relatório	Indicador 14 = tipo de produção: atendimento individual; problema/condição avaliada: pré-natal, puericultura, puerpério (até 42 dias), saúde sexual e reprodutiva, tabagismo, usuário de álcool, usuário de outras drogas, saúde mental, doenças transmissíveis: dengue; DST; hanseníase, tuberculose; rastreamento câncer de mama, do colo do útero, rastreamento risco cardiovascular
	Indicador 15 = tipo de produção: atendimento individual; problema/condição avaliada: idem ao 14; CIAP/CID: CIAP (50) medicação/prescrição/renovação/injeção, CID (Z760) emissão de e prescrição de repetição
	Indicador 16 = tipo de produção: procedimento; procedimento: teste rápido dosagem de proteinúria, teste rápido de HIV, teste rápido de gravidez, teste rápido para hepatite C, teste rápido para sífilis e triagem oftalmológica
	Indicador 17 = tipo de produção: atendimento individual; problema/condição avaliada: idem ao 14; conduta: agendamento p/ NASF*, agendamento para grupos, alta do episódio, encaminhamento interno no dia, encaminhamento intersetorial, encaminhamento para CAPS, encaminhamento p/ internação hospitalar, encaminhamento para serviço de atenção domiciliar, encaminhamento para serviço especializado, encaminhamento para urgência, retorno para cuidado continuado/programado, retorno para consulta agendada
Tipo de cuidado	Consulta agendada. prog/cuid. cont., Consulta agendada, Dem. esp. esc. inicial/orient., Dem. esp. consulta no dia, Dem. esp. atendimento urgência
Categoria profissional	Enfermeiro
Setor de cuidado	UBS, Unidade móvel, Rua, Domicílio, Escola/creche, Outros, Polo (academia da saúde), Instituição/abrigo, Unidade prisional ou congêneres, Unidade socioeducativa
Tipo de equipe de saúde	Equipe de Saúde da Família - ESF, Equipe Agente Comunitário de Saúde - EACS, NASF*/eMulti, Equipe da Atenção Básica - EAB, Equipe Consultório na Rua - ECR, Equipe de Saúde Bucal - SB, Equipe AB Prisional - EABp, Equipe de Atenção Primária - eAP.

*NASF = Núcleo de Apoio à Saúde da Família

Reconhece-se que esses indicadores correspondem a registros administrativos de produção, e não ao detalhamento da complexidade clínica envolvida em cada ação. Ainda assim, representam dimensões estruturantes do escopo legal e normativo da prática do enfermeiro na APS, permitindo analisar a distribuição territorial dessas atividades e sua aproximação com competências descritas no referencial internacional. 

### Procedimentos de coleta

A extração dos dados do SISAB foi realizada entre os dias 3 e 5 de novembro de 2024, por meio de processo automatizado e parametrizado, com aplicação de um único filtro padronizado para todos os estados, modalidades de equipe e unidades de atendimento, conforme as opções estruturais disponibilizadas pelo próprio sistema. Foram selecionados registros referentes aos 12 meses de 2023, restringindo-se a profissionais da categoria enfermeiro e abrangendo as diferentes modalidades habilitadas na APS [Equipe de Saúde da Família (ESF), Equipe de Atenção Primária (eAP), Equipe Consultório de Rua (eCR) e demais equipes reconhecidas], de acordo com os códigos e parâmetros padronizados do Sistema de Gerenciamento da Tabela de Procedimentos, Medicamentos e OPM (SIGTAP) do Sistema Único de Saúde (SUS), conforme descrito na Figura 1. Esse procedimento assegurou uniformidade à seleção e reduziu a heterogeneidade decorrente de diferenças de codificação entre regiões e tipos de equipe.

Após a extração, o banco de dados passou por validação dupla conduzida por um estatístico e por um segundo pesquisador responsável pela conferência e manipulação do banco, garantindo integridade, consistência e aplicabilidade dos registros. Por se tratar de dados agregados por atendimento, data e procedimento, segundo os parâmetros oficiais do SISAB, não houve ocorrência de duplicidade dentro dos critérios estabelecidos. Registros incompletos, inválidos ou incompatíveis com a APS foram automaticamente excluídos pelo filtro aplicado durante a extração.

Adicionalmente, dados geoespaciais (mapas e coordenadas geográficas) foram obtidos no site do Instituto Brasileiro de Geografia e Estatística (IBGE)^19^, utilizando-se projeção geográfica. Foram compiladas as informações relacionadas ao tamanho da população geral para cada unidade da federação e o total da população brasileira (ano censitário). Os mapas digitais e respectivas coordenadas geodésicas (arquivos nos formatos *.*shp*, *.*shx* e *.*prj*) do território nacional e sua subdivisão em unidades da federação (referência *loop* 2017) estão disponíveis em http:downloads.ibge.gov.br/downloads_geociencias.htm. Os arquivos com extensão .*shp* assumem a projeção geográfica SIRGAS_2000, *datum*[“d_SIRGAS_2000”, *spheroid*[“grs_1980”,6378137,298.257222101], *primem*[“*Greenwich*”,0], *unit*[“*degree*”, 0.017453292519943295]].

### Análise dos dados

Essa etapa foi dividida em duas fases.

#### Fase 1 - Análise descritiva

Foram quantificadas as frequências absolutas das atividades associadas aos indicadores selecionados, por unidade da federação. Essa fase permitiu estimar o volume de ações realizadas por enfermeiros no âmbito da APS, classificadas conforme os domínios da prática avançada (Figura 2). Utilizou-se ainda, como referência, a força de trabalho de enfermeiros vinculados à APS registrada no Cadastro Nacional de Estabelecimentos de Saúde - CNES/Profissionais^17^. A extração foi realizada com base nos dados disponíveis para o mês de julho de 2023, totalizando 100.614 enfermeiros em atividade na APS, no país. 

**Figura 2:** Frequência absoluta de Enfermeiros e de intervenções, em termos de consulta, gerenciamento de episódios complexos de cuidados, autoridade para diagnosticar, prescrever medicamentos, testes diagnósticos e tratamentos terapêuticos, nas 26 Unidades da Federação do país e Distrito Federal. Brasil, 2023

UF	Estado	Número de Enfermeiros	Indicador 14^*^	Indicador 15^†^	Indicador 16^‡^	Indicador 17^§^
1	Acre	477	304027	4	128971	371863
2	Alagoas	1812	1035600	213	251660	1904659
3	Amapá	556	93576	178	31724	189465
4	Amazonas	1627	1346253	259	452464	2059683
5	Bahia	6604	3873064	1285	583719	6076458
6	Ceará	4582	2963851	1190	450512	5990449
7	Distrito Federal	1618	601603	45	88536	887839
8	Espírito Santo	1861	983349	4666	131563	1586019
9	Goiás	2985	1481209	3664	220240	2538890
10	Maranhão	3686	1940515	650	518498	3427834
11	Mato Grosso	1673	1012717	153	226878	1606304
12	Mato Grosso do Sul	1326	722841	40	178672	1113670
13	Minas Gerais	11927	4975276	3876	771176	9215929
14	Pará	3456	1753622	442	378462	2772583
15	Paraíba	2474	1244622	601	121172	2229152
16	Paraná	6431	3405789	2409	591524	4931203
17	Pernambuco	4148	2983259	165	366765	4587135
18	Piauí	2361	950709	17	48363	1906376
19	Rio de Janeiro	5726	3858600	5765	653215	5390978
20	Rio Grande do Norte	2033	1023808	172	147694	1574712
21	Rio Grande do Sul	6707	3578698	3161	916419	5090050
22	Rondônia	794	372872	46	139102	480213
23	Roraima	381	105395	0	58952	160319
24	Santa Catarina	4842	2541553	1260	735644	3936650
25	São Paulo	18216	7913901	6523	1141077	11242152
26	Sergipe	1236	640694	264	120886	947929
27	Tocantins	1075	421043	164	58906	807368
	Total Geral	100.614	52.128.446	37.212	9.512.794	83.025.882

*Indicador 14 = Consultas; ^†^Indicador 15 = Prescrições de medicamentos; ^‡^Indicador 16 = Prescrições de testes; ^§^Indicador 17 = Encaminhamentos

#### Fase 2 - Cálculo de indicadores e georreferenciamento

A partir das informações totalizadas (Figura 2), foram construídos dois índices.

1) Proporção ajustada: calculada via regressão linear múltipla, com ajuste para o tamanho da população de enfermeiros. Essa técnica foi utilizada para se obter o número esperado de ações de cada um dos indicadores avaliados, levando-se em consideração o tamanho da população de enfermeiros (covariada) de cada unidade da federação, com o objetivo de minimizar o efeito de confundimento da distribuição desigual desses profissionais no território nacional. O índice foi calculado de acordo com a equação:

$$Proporção\;ajustada=\frac{N\;esperado\;do\;Indicador\;do\;Figura\;2}{total\;geral\;do\;Indicador}*100$$

A qualidade de ajuste dos modelos de regressão linear foi avaliada a partir do R^2^ e R^2^ ajustado (ambos superiores a 90% nos quatro modelos) e da homocedasticidade dos resíduos (dados não apresentados). Este índice permite a comparação entre os Indicadores da Figura 2, *intra* unidades da federação.

2) Taxa padronizada: estimada por padronização direta^15^, adotando-se o total da população brasileira (ano censitário) como população padrão. A partir das totalizações apresentadas na Figura 2, calcularam-se as proporções brutas [(N observado do Indicador/total geral do Indicador)] e estimaram-se os índices de acordo com a equação:

$$Taxa\;padronizada=\frac{proporção\;bruta\;do\;Indicador}{população\;padrão}*\frac{10^{n}\;habitantes}{ano}$$

Esse índice permite a comparação em duas direções: *intra* e *entre* unidades da federação. Para a plotagem em mapas temáticos^20^, esse índice foi classificado em quartos, de acordo com os quartis da distribuição. 

Todas as análises foram efetuadas no *software* Stata^®^, versão 13.2, com aplicação do pacote de comandos *spmap* ^21^ para a etapa de georreferenciamento das taxas padronizadas. 

### Considerações éticas

Por se tratar de estudo baseado exclusivamente em dados secundários, agregados e de domínio público, não foi necessária aprovação por comitê de ética, conforme dispensa prevista na Resolução CNS nº 510/2016.

## Resultados

A Tabela 1 apresenta a distribuição proporcional ajustada das principais ações realizadas por enfermeiros na APS, considerando o exercício de competências compatíveis com a PAE. Em todas as UFs, o indicador que mais se destacou em magnitude foi o 16, ou seja, autoridade para prescrever testes diagnósticos e tratamentos terapêuticos, com exceção dos estados do Rio de Janeiro e de São Paulo.

**Tabela 1:** Proporções ajustadas dos Indicadores, segundo as unidades da federação. Brasil, 2023

Unidades Federativas	Proporções (%) Ajustadas*			
	Indicador 14	Indicador 15	Indicador 16	Indicador 17
Acre	0,7	0,3	1,0	0,8
Amapá	0,8	0,4	1,1	0,8
Amazonas	2,4	2,0	2,6	2,4
Pará	3,0	2,7	3,2	3,0
Rondônia	1,1	0,8	1,4	1,2
Roraima	0,7	0,3	1,0	0,7
Tocantins	1,4	1,0	1,6	1,4
Alagoas	1,8	1,4	2,0	1,8
Bahia	6,6	6,5	6,7	6,6
Ceará	4,5	4,2	4,6	4,5
Maranhão	3,0	2,7	3,1	2,9
Paraíba	2,7	2,3	2,9	2,7
Pernambuco	5,1	4,9	5,3	5,1
Piauí	1,9	1,6	2,2	1,9
Rio Grande do Norte	1,9	1,6	2,2	1,9
Sergipe	1,5	1,2	1,8	1,6
Espírito Santo	2,0	1,7	2,3	2,0
Minas Gerais	8,8	8,7	8,8	8,7
Rio de Janeiro	9,1	9,1	9,1	9,1
São Paulo	24,0	24,7	23,5	24,0
Paraná	5,0	4,8	5,1	5,0
Rio Grande do Sul	4,6	4,4	4,8	4,6
Santa Catarina	3,2	2,9	3,4	3,2
Distrito Federal	3,1	2,8	3,3	3,1
Goiás	3,2	2,9	3,4	3,2
Mato Grosso	2,0	1,7	2,3	2,0
Mato Grosso do Sul	1,6	1,3	1,9	1,7

*Ajuste para o tamanho da população de enfermeiros

A Tabela 2 exibe as taxas padronizadas dos indicadores, segundo as unidades da federação. Comparações inter-regionais revelam que São Paulo lidera os valores em todos os indicadores (variação entre 5,6 e 8,2), seguido por Minas Gerais, Rio de Janeiro e Paraná. 

**Tabela 2:** Distribuição das taxas padronizadas, segundo as unidades da federação. Brasil, 2023

Unidades Federativas	Taxas Padronizadas*			
	Indicador 14	Indicador 15	Indicador 16	Indicador 17
Acre	0,3	0	0,6	0,2
Amapá	0,1	0,2	0,2	0,1
Amazonas	1,2	0,3	2,2	1,2
Pará	1,6	0,6	1,9	1,6
Rondônia	0,3	0,1	0,7	0,3
Roraima	0	0	0,3	0,1
Tocantins	0,4	0,2	0,3	0,5
Alagoas	0,9	0,3	1,2	1,1
Bahia	3,5	1,6	2,9	3,4
Ceará	2,7	1,5	2,2	3,4
Maranhão	1,7	0,8	2,6	1,9
Paraíba	1,1	0,7	0,6	1,3
Pernambuco	2,7	0,2	1,8	2,6
Piauí	0,8	0	0,2	1,1
Rio Grande do Norte	0,9	0,2	0,7	0,9
Sergipe	0,6	0,3	0,6	0,5
Espírito Santo	0,9	5,9	0,6	0,9
Minas Gerais	4,5	4,9	3,8	5,2
Rio de Janeiro	3,5	7,3	3,2	3,0
São Paulo	7,1	8,2	5,6	6,4
Paraná	3,1	3,0	2,9	2,8
Rio Grande do Sul	3,2	4,0	4,5	2,9
Santa Catarina	2,3	1,6	3,6	2,2
Distrito Federal	0,5	0,1	0,4	0,5
Goiás	1,3	4,6	1,1	1,4
Mato Grosso	0,9	0,2	1,1	0,9
Mato Grosso do Sul	0,6	0,1	0,9	0,6

*Padronização pelo método direto, população padrão: total da população brasileira

A Figura 3 apresenta graficamente a espacialização das taxas padronizadas em quartis, permitindo a visualização geográfica das ações compatíveis com PAE na APS. Observa-se um padrão de maior concentração dos indicadores nos estados que compõem as regiões Sudeste e Sul, com destaque para São Paulo, Minas Gerais, Rio de Janeiro, Paraná, Rio Grande do Sul e Santa Catarina. Em contraposição, os estados da Região Norte e parte do Nordeste apresentam menores concentrações de indicadores de desempenho de PAE. A visualização evidencia um gradiente geográfico de desigualdade no exercício da autonomia e autoridade clínica dos enfermeiros. Esse padrão sugere a existência de contextos mais favoráveis à consolidação da PAE, e de outros menos favoráveis, em que as práticas clínicas precisam ser revistas e expandidas, desde que sustentadas por protocolos de regulação municipais e investimentos estruturantes em formação especializada e desenvolvimento profissional contínuo. 

**Figura 3:** Distribuição geográfica, por unidade da federação, dos indicadores de consulta, gerenciamento de episódios complexos de cuidados, autoridade para diagnosticar, prescrever medicamentos, testes diagnósticos e tratamentos terapêuticos realizados por enfermeiros na Atenção Primária à Saúde. Brasil, 2023

## Discussão

As ações realizadas pelo enfermeiro na APS convergem para as diretrizes estabelecidas pelo ICN^8^ para a prática avançada de enfermagem. Este estudo concentrou-se na análise de ações que traduzem autoridade e autonomia clínica do enfermeiro: realização de consultas, encaminhamentos clínicos, prescrição de testes diagnósticos e prescrição de medicamentos no âmbito da APS.

A distribuição desses quatro conjuntos de ações, em termos de volume de registros, ocorreu na seguinte ordem: consulta de enfermagem, encaminhamento de usuários para outros profissionais e/ou serviços de saúde, prescrição de testes diagnósticos e, por fim, prescrição de medicamentos. Como estudo ecológico, as inferências dizem respeito às unidades coletivas, e não ao desempenho individual dos profissionais. A possibilidade de vieses relacionados à qualidade do registro e à heterogeneidade estrutural dos serviços deve ser considerada, embora medidas de padronização tenham sido adotadas.

A Figura 3 é central para a compreensão do padrão geográfico de implementação das quatro modalidades de ação alinhadas com a PAE no Brasil. A imagem evidencia uma concentração dos quatro indicadores no eixo Sul-Sudeste, sugerindo maior institucionalização das práticas avançadas nesses territórios. Por outro lado, estados da Região Norte e Nordeste, como Acre, Roraima, Piauí e Amapá, situam-se nos quartis inferiores, indicando menor densidade de ações clínicas compatíveis com a PAE, o que pode refletir limitações estruturais, escassez de protocolos locais ou subnotificação. Esse gradiente evidencia que, embora as ações desenvolvidas no Brasil por enfermeiros na APS apresentem alinhamento com os pressupostos da PAE, persistem desigualdades regionais significativas entre os estados da federação. Assim sendo, a expansão da PAE parece estar dependente de investimentos no âmbito da capacitação profissional, normatização local e financiamento público.

O expressivo número de consultas de enfermagem registradas (52.128.446) demonstrou a ampla atuação clínica dos enfermeiros, especialmente em condições que requerem maior autonomia diagnóstica, tais como, a saúde da mulher, a saúde mental, as doenças transmissíveis e o rastreamento de câncer. Essa prática, embora já consolidada em parte dos territórios, apresentou distribuição desigual entre as Unidades da Federação, com destaque para São Paulo, Minas Gerais, Rio de Janeiro, Paraná, Bahia e Rio Grande do Sul, conforme identificado na Figura 3 (quartil superior da análise espacial). Esses achados reforçam a existência de contextos mais favoráveis à consolidação da PAE nesses estados.

Estudos nacionais corroboram esse cenário ao destacarem a percepção positiva da autonomia do enfermeiro na APS^14^ ^),(^ ^22^ ^)-(^ ^24^ e evidenciarem que, quando respaldados por normativas e evidência científica, esses profissionais assumem um papel protagonista no cuidado clínico, sobretudo em áreas sensíveis, como as Infeções Sexualmente Transmissíveis (ISTs) e a atenção à saúde da mulher^25^. 

Assim, os resultados apresentados neste estudo não correspondem a um modelo formalmente reconhecido de Prática Avançada de Enfermagem no Brasil, dado que essa função carece de regulamentação específica, mas evidenciam dimensões do exercício clínico do enfermeiro que se alinham a competências descritas em referenciais internacionais e apontam potencial de desenvolvimento. Essas ações, realizadas por enfermeiros generalistas no âmbito do escopo ampliado previsto na legislação brasileira, revelam padrões de maturidade clínica e protagonismo que podem subsidiar a construção de marcos formativos e regulatórios alinhados às diretrizes internacionais da PAE. Os resultados demonstram um conjunto expressivo de práticas que dialogam com domínios avançados, oferecendo base empírica para orientar debates e propostas futuras.

Além disso, foram identificados 83.025.882 registros de encaminhamentos, evidenciando o papel do enfermeiro como coordenador do cuidado e articulador de fluxos assistenciais. A análise por quartis mostrou maior concentração desses registros nos estados do Ceará, Bahia, Minas Gerais, São Paulo, Rio de Janeiro e Rio Grande do Sul, conforme representado na Figura 3. Essa concentração pode estar relacionada à maior institucionalização de protocolos clínicos municipais que conferem respaldo às decisões clínicas dos enfermeiros. A literatura^26^ corrobora esse achado ao destacar o papel do “enfermeiro de ligação” como elemento-chave na articulação efetiva das redes de atenção à saúde, especialmente em processos de referência e contrarreferência.

No que se refere à prescrição de testes diagnósticos e tratamentos terapêuticos (n=9.512.794), os resultados indicaram maior expressividade nos estados de Minas Gerais, Rio de Janeiro, São Paulo, Paraná, Santa Catarina e Rio Grande do Sul, sugerindo melhores condições estruturais e organizacionais, como maior cobertura da Estratégia Saúde da Família (ESF) e capacitação técnica. 

Por outro lado, os resultados referentes à prescrição de medicamentos por enfermeiros (n=37.212) foram relativamente baixos em comparação com o volume de consultas e encaminhamentos. Embora a PNAB autorize a prescrição por enfermeiros, a efetivação dessa competência depende de protocolos locais, o que gera disparidades entre estados. A Figura 3 evidencia maior incidência de prescrições nos estados de São Paulo, Rio de Janeiro, Espírito Santo, Minas Gerais, Goiás e Paraná. Estudos anteriores^9^ ^),(^ ^14^ ^),(^ ^27^ ^)^ indicam que os enfermeiros se sentem preparados e reconhecem a prescrição como um avanço da autonomia profissional, desde que respaldada por normativas técnicas. Portanto, os baixos números nacionais relativos à prescrição por enfermeiros podem estar relacionados à inexistência ou à desatualização de protocolos municipais, insegurança jurídica e barreiras institucionais. É de realçar que a prescrição de medicamentos, no contexto da Prática Avançada de Enfermagem na APS, representa um avanço alinhado aos princípios da integralidade e da resolutividade do cuidado^9^ ^),(^ ^14^. 

Nesse contexto, o ICN recomenda a inclusão da prescrição de medicamentos no escopo de ação da PAE, em nível global, desde que acompanhada de formação e regulação específica, bem como de suporte institucional^8^. 

Na Europa, a implementação de PAE apresenta-se de forma heterogênea, sendo que apenas 11 dos 35 países da região pan-europeia possuem legislação formal sobre o tema^28^. Ainda prevalece grande variabilidade na definição de PAE, especialmente no que se refere a nomenclatura, escopo de ação, regulação, exigências educacionais para o exercício da função e estratégias de financiamento^28^, o que compromete a sua concretização. Em países como a França, por exemplo, a ambiguidade de papéis, aliada à indefinição quanto ao modelo de financiamento e ao apoio institucional, condiciona o desenvolvimento da PAE, cenário que também se verifica em países, tais como a Inglaterra, onde a prática se encontra amplamente estabelecida^29^ ^)-(^ ^30^. Em países nórdicos e da Oceania, como Austrália, Finlândia e Noruega, há maior padronização na formação, regulação e reconhecimento da PAE como sendo estratégica para os sistemas de saúde^31^. Estudo europeu ressalta a importância de se combater o isolamento profissional, por meio de redes de contato e mentoria, como estratégias essenciais para promover a sustentabilidade da força de trabalho de enfermagem de prática avançada^32^. Na região das Américas, 64% dos países implementaram funções de enfermagem avançada^33^, contudo, os países da América Latina devem acentuar o investimento nessa estratégia^14^ ^),(^ ^34^, valorizando o diálogo entre as partes interessadas^35^.

 Nos contextos em que a discussão sobre PAE ainda é incipiente, embora considere uma iniciativa promissora^36^ ^)-(^ ^37^, torna-se essencial fortalecer a base educacional, política e institucional da enfermagem para garantir a introdução sustentável e efetiva da prática avançada^38^. Adicionalmente, a educação continuada foi considerada um fator-chave para fomentar a confiança no desempenho da função de enfermagem de prática avançada, conforme demonstrado em estudos cujo foco foi a análise do tipo de intervenção educacional ao longo do tempo de implementação^39^. No caso brasileiro, é justamente a base educacional que deve se tornar o foco das ações estratégicas das lideranças da enfermagem, uma vez que, ainda que se observe um número crescente de intervenções clínicas avançadas na APS, a ausência de normativas nacionais específicas, somada à dependência de regulamentação local, compromete a equidade no exercício da PAE entre os estados. O estudo também apontou que existe massa crítica instalada para avançar: com mais de 100 mil enfermeiros atuando na APS, a que se deve associar um robusto sistema de formação em enfermagem (cursos de graduação, mestrados e doutorados acadêmicos e profissionais)^40^ e lideranças organizadas em especialidades e sociedades científicas. 

Apesar da abrangência nacional e da robustez dos dados utilizados, este estudo apresenta algumas limitações que devem ser consideradas. A principal delas refere-se à possível subnotificação das ações registradas nos sistemas oficiais de informação em saúde, especialmente nas regiões Norte e Nordeste, como observado nos estados do Piauí, Roraima e Acre, onde a magnitude dos indicadores padronizados foi igual a zero ou extremamente baixa. Tal cenário pode não refletir a inexistência das práticas clínicas compatíveis com a Prática Avançada de Enfermagem (PAE), mas sim fragilidades no registro, inconsistências nos sistemas de informação ou ausência de codificação padronizada nas unidades de saúde. Além disso, a natureza ecológica e agregada da análise impede inferências causais ou interpretações individualizadas. Assim, são necessários estudos adicionais que forneçam informação desagregada.

Importa ainda destacar que as ações analisadas não são, por si mesmas, equivalentes à PAE. No contexto brasileiro, muitas delas integram o escopo ampliado do enfermeiro generalista no SUS, conforme previsto na PNAB e na lei do exercício profissional, e não implicam necessariamente competências avançadas como definidas pelo ICN. 

Ainda assim, a execução consistente e disseminada das ações analisadas neste estudo evidencia a maturidade clínica que os enfermeiros vêm desenvolvendo na APS, demonstrando capacidade técnica, responsabilização e protagonismo no cuidado. Esses achados revelam, além disso, um potencial significativo para orientar políticas de formação, regulação e ampliação do escopo profissional, ao oferecerem dados concretos sobre a atuação real da enfermagem no território nacional. Assim, os indicadores analisados fundamentam, de maneira clara, o papel central dos enfermeiros na APS brasileira e contribuem para debates estratégicos sobre a regulamentação e o fortalecimento da prática avançada no país, sem, contudo, caracterizar diretamente tais ações como Prática Avançada de Enfermagem, mas evidenciando dimensões clínicas que dialogam com esse referencial internacional.

## Conclusão

Este estudo ecológico oferece uma visão nacional da distribuição e intensidade das ações clínicas realizadas por enfermeiros na Atenção Primária à Saúde. Embora não permita inferir a complexidade do raciocínio clínico ou caracterizar competências avançadas nos termos definidos pelo ICN, os padrões observados revelam um campo de prática robusto, no qual a enfermagem desempenha funções centrais no cuidado e acumula experiência que pode sustentar avanços estruturantes.

A heterogeneidade territorial identificada indica a influência de capacidades organizacionais, infraestrutura e disponibilidade de profissionais, evidenciando áreas que demandam fortalecimento e qualificação. Além disso, a presença de mais de 100 mil enfermeiros atuando na APS, associada a uma sólida estrutura formativa com massa crítica consolidada nos níveis de pós-graduação, acadêmico e profissional, configura um cenário estratégico para o desenvolvimento de programas de formação avançada, inclusive Mestrados Profissionais orientados à prática clínica ampliada e alinhados às necessidades do SUS.

Os achados apontam caminhos concretos para aprimorar o exercício clínico da enfermagem no país, incluindo a qualificação dos sistemas de registro, a padronização de codificação, a harmonização de protocolos e a realização de estudos qualitativos e de métodos mistos que aprofundem a compreensão dos processos decisórios e das condições que moldam a autonomia profissional na APS. Ao oferecer evidências empíricas sobre o trabalho clínico dos enfermeiros em escala nacional, este estudo contribui para debates estratégicos sobre o fortalecimento e a ampliação do escopo clínico, reforçando a relevância de medidas que avancem na direção da regulamentação e valorização da autonomia e autoridade clínica da enfermagem, elementos centrais para a efetividade e sustentabilidade da Atenção Primária no Brasil.

## Data Availability

Todos os dados gerados ou analisados durante este estudo estão incluídos neste artigo publicado.

# Translation: Acciones del enfermero indicativas de autonomía y autoridad compatibles con la Enfermería de Práctica Avanzada brasileña*

**DOI:** 10.1590/1518-8345.8245.4954

**Authors:** Emerson Willian Santos de Almeida, Simone de Godoy, Maria Alice Barbosa Fortunato, Elaine Maria Leite Rangel Andrade, Ítalo Rodolfo Silva, Patrícia Sofia Oliveira Fonseca Nunes, Isabel Amélia Costa Mendes

**Affiliations:** 1 Universidade de São Paulo, Escola de Enfermagem de Ribeirão Preto, Centro Colaborador de la OPS/OMS para el Desarrollo de la Investigación en Enfermería, Ribeirão Preto, SP, Brasil.; 2 Organização Mundial da Saúde - África, WHO - AFRO, Guinea-Bissau.; 3 Universidade Federal do Piauí, Ininga, Teresina, PI, Brasil.; 4 Universidade Federal do Rio de Janeiro, Rio de Janeiro, RJ, Brasil.; 5 Universidade dos Açores, Escola Superior de Saúde, Angra do Heroísmo, Açores, Portugal.

**Keywords:** Atención Primaria de Salud, Autonomía Profesional, Enfermería, Estudio Ecológico, Práctica Avanzada de Enfermería, Políticas de Salud

## Abstract

**Objetivo:**

este estudio analizó acciones representativas de la autonomía y la autoridad clínica de los enfermeros en la atención primaria de salud, compatibles con la Enfermería de Práctica Avanzada en Brasil.

**Método:**

estudio ecológico con datos secundarios de 2023 del sistema de información del Ministerio de Salud de Brasil, considerando los 26 estados y el Distrito Federal. Se examinaron indicadores de consultas, prescripciones de medicamentos, pruebas diagnósticas y derivaciones clínicas.

**Resultados:**

se evidenció una amplia actuación de los enfermeros en ámbitos característicos de la Enfermería de Práctica Avanzada, aunque con marcada desigualdad geográfica, predominante en el Sudeste y Sur.

**Conclusión:**

el análisis revela el ejercicio de competencias avanzadas, incluso sin regulación específica, y destaca la necesidad de políticas que promuevan la formación especializada, la estandarización normativa y el reconocimiento legal de la Enfermería de Práctica Avanzada, con el objetivo de mejorar la calidad y equidad de la atención en salud.

## Destacados

**(1)** El resultado sostiene que la Enfermería de Práctica Avanzada ya se concreta en el día a día de los servicios.

**(2)** 52 millones de consultas de enfermería; 37.000 prescripciones de medicamentos.

**(3)** 9,5 millones de pruebas diagnósticas prescritas y 83 millones de derivaciones clínicas.

**(4)** Ejercicio efectivo de acciones avanzadas, incluso sin regulación específica.

**(5)** Terreno fértil para la institución de maestrías profesionales en la Enfermería de Práctica Avanzada, alineadas con el SUS y el ICN.

## Introducción

La reconfiguración de la fuerza laboral en el sector de la salud se ha intensificado ante la sobrecarga de los servicios, la insuficiencia de recursos, la escasez de profesionales y el aumento de la demanda en un contexto de envejecimiento de la población, crecimiento de las enfermedades crónicas y pandemias recientes. El avance hacia la cobertura sanitaria universal requiere no solo el fortalecimiento de los sistemas de salud, sino también la movilización de una fuerza laboral cualificada, con capacidad para responder a las complejas necesidades de la población^1^ ^)-(^ ^2^.

Los enfermeros están desempeñando un papel cada vez más estratégico, sobre todo en los niveles primarios de atención en salud, en los que la integralidad y la longitudinalidad de la atención son fundamentales^3^.

La valorización de la profesión, mediante la inversión en formación, regulación y ampliación del ámbito de práctica, ha sido señalada por organismos internacionales como un eje esencial para alcanzar los Objetivos de Desarrollo Sostenible (ODS) y la equidad en salud. El informe *State of the World’s Nursing* 2025, de la Organización Mundial de la Salud (OMS), destaca la importancia estratégica de la enfermería para alcanzar la cobertura sanitaria universal y recomienda, entre otras medidas, el fortalecimiento de la formación profesional y la adopción de la Enfermería de Práctica Avanzada (EPA) como respuesta a las crecientes demandas de los sistemas de salud^1^. En este sentido, las evidencias demuestran que los enfermeros de práctica avanzada pueden alcanzar resultados clínicos comparables a los de los médicos, con altos niveles de satisfacción entre los pacientes, especialmente en la atención primaria de salud (APS)^4^.

Aunque la EPA se originó en los Estados Unidos en la década de 1960, el concepto se fue desarrollando y ganando relevancia en países como Canadá, Reino Unido y Australia a finales del siglo XX, y más recientemente se ha extendido a países de ingresos bajos y medios. Su adopción internacional se ha producido de forma gradual y desigual en cada país y región, marcada por retos persistentes, como la ambigüedad de funciones, los obstáculos legales y la resistencia institucional^5^ ^)-(^ ^6^.

A nivel internacional, las revisiones indican que la transición de los enfermeros a funciones avanzadas en la APS se ve afectada por factores como la claridad del alcance, el apoyo de las partes interesadas y la calidad de las relaciones interprofesionales^7^ ^)-(^ ^8^. Además, considerada un modelo prometedor para ampliar el acceso y la resolutividad de los servicios, inicialmente concebida para zonas con escasez de médicos y alta demanda de cuidados continuados, la autoridad para prescribir medicamentos ocupa un lugar central, ya que representa tanto una ampliación del ámbito clínico como un hito simbólico de reconocimiento profesional^8^, entre otras competencias que caracterizan la actuación de los enfermeros de práctica avanzada.

El Consejo Internacional de Enfermeras (ICN, según su acrónimo en inglés)^8^ define la EPA como un nivel ampliado de práctica caracterizado por competencias clínicas avanzadas, juicio diagnóstico más complejo, toma de decisiones autónoma y autoridad para prescribir, solicitar exámenes y realizar derivaciones^8^ ^)-(^ ^9^. Estas competencias se basan en requisitos específicos de formación y regulación, y el ICN recomienda que los enfermeros de práctica avanzada tengan, como mínimo, un título de maestría, en programas formalmente reconocidos, con planes de estudio que incluyan disciplinas centrales como fisiopatología avanzada, farmacología, examen físico avanzado y razonamiento diagnóstico, además de una sólida carga horaria clínica orientada al manejo de condiciones complejas y la toma de decisiones independientes^8^ ^)-(^ ^11^.

Aunque no existe una normativa específica para la EPA en Brasil, la Política Nacional de Atención Básica (PNAB)^12^ asigna a los enfermeros actividades que dialogan directamente con estos ámbitos internacionales, tales como la consulta de enfermería, la evaluación diagnóstica, la solicitud de exámenes, la prescripción de medicamentos en protocolos y la derivación. Asociadas al respaldo de la ley del ejercicio profesional y de las normativas del consejo de clase^13^, estas atribuciones configuran un ámbito clínico ampliado, que presenta intersecciones concretas con las competencias descritas por el ICN^8^, aunque carezca de titulación formal en prácticas avanzadas y de un marco regulatorio específico para esta categoría profesional^14^.

Las acciones representativas de la autonomía y la autoridad del enfermero, compatibles con el ejercicio de la EPA, han sido poco exploradas en la literatura brasileña^14^. Teniendo en cuenta el potencial de la EPA para fortalecer los sistemas de salud y la creciente demanda de los enfermeros de una mayor autonomía clínica, es esencial analizar cómo se está manifestando esta práctica en contextos específicos, como el brasileño. De este modo, se habla de autonomía a partir de las bases legales y deontológicas que deben orientar el alcance de las prácticas avanzadas de los enfermeros en Brasil. Por su parte, la autoridad del enfermero está relacionada con el conjunto de competencias que implican las EPA, especialmente en lo que se refiere al conjunto de conocimientos y habilidades orientados por las mejores evidencias científicas, debidamente contextualizadas en el tiempo y el espacio, es decir, de acuerdo con el perfil de los pacientes, las tecnologías disponibles y la experiencia del profesional; por lo tanto, la autoridad clínica se refiere a la prerrogativa de prescribir, solicitar exámenes y realizar derivaciones, competencias históricamente reconocidas como dominios avanzados en modelos internacionales de práctica avanzada y ejercidas en Brasil de forma regulada por protocolos y normas profesionales. 

Por lo tanto, en este estudio, la autonomía clínica se entiende como la capacidad del enfermero para realizar consultas, realizar evaluaciones diagnósticas y tomar decisiones durante el proceso de atención, tal como se establece en las directrices nacionales. Por lo tanto, investigar aspectos clave, como la autoridad diagnóstica y las responsabilidades prescriptivas en el contexto de la APS, puede contribuir a identificar qué competencias clínicas ejercen con mayor frecuencia los enfermeros, incluso en ausencia de una regulación de la EPA. Estos datos pueden ser fundamentales para respaldar, entre otras cosas, la actualización de las Directrices Curriculares Nacionales para la licenciatura en Enfermería, así como para mejorar los programas de posgrado y alinear la formación profesional con las demandas reales de la APS. Además, este análisis puede fortalecer la base empírica necesaria para la consolidación de la EPA en Brasil, promoviendo un mayor reconocimiento, valoración y autonomía de la enfermería.

El objetivo de este estudio fue analizar acciones representativas de la autonomía y la autoridad clínica de los enfermeros en la atención primaria de salud, compatibles con la Enfermería de Práctica Avanzada en Brasil.

## Método

### Diseño del estudio

Se trata de un estudio ecológico^15^, con unidad de análisis agregada, que comprende el territorio brasileño y su subdivisión en 26 estados y el Distrito Federal de Brasil, en el año 2023.

### Fuentes secundarias de datos y variables

Se utilizaron bases de datos públicas secundarias, extraídas de las siguientes plataformas nacionales: Sistema de Información en Salud para la Atención Básica (SISAB)^16^ y Registro Nacional de Establecimientos de Salud (CNES/Profesionales)^17^. 

A partir de estas dos bases de datos se clasificaron cuatro indicadores (14, 15, 16 y 17) relacionados con las competencias de la EPA y la selección, extracción y organización de los datos siguieron una matriz conceptual previamente estructurada, derivada de un estudio anterior^18^, que establece la relación entre las competencias de la EPA, según lo propuesto por el ICN^8^ y las disposiciones normativas de la PNAB^12^ brasileña. La matriz conceptual utilizada en el estudio permitió establecer la correspondencia entre las competencias clínicas reconocidas internacionalmente y las atribuciones conferidas a los enfermeros por la ley del ejercicio profesional y por la PNAB^12^, que, mediante protocolos y regulaciones municipales, autorizan la realización de consultas, la solicitud de exámenes, la prescripción de medicamentos y la realización de derivaciones. 

A efectos analíticos, se adoptaron definiciones operativas sintéticas: autonomía clínica como la capacidad del enfermero para realizar consultas y evaluaciones diagnósticas; y autoridad clínica como la prerrogativa de prescribir, solicitar exámenes y derivar a los usuarios de acuerdo con los marcos normativos vigentes. Sobre la base de estas definiciones, los indicadores se clasificaron según su naturaleza clínica: indicador 14 (consultas), que representa la autonomía clínica en las diferentes áreas previstas en el SISAB^16^, como atención prenatal, puericultura, puerperio, salud sexual y reproductiva, salud mental, tabaquismo, consumo de alcohol y otras drogas, seguimiento de enfermedades transmisibles y acciones de tamizaje; indicador 15 (solicitud de exámenes), que expresa la autoridad diagnóstica; indicador 16 (prescripción de medicamentos), que refleja la autoridad terapéutica; e indicador 17 (derivaciones), que indica una mayor autoridad clínica en la derivación del usuario a otros puntos de la red asistencial. Los cuatro indicadores representan dimensiones clínicas avanzadas, tal y como se detalla en la Figura 1.

**Figura 1:** Filtros utilizados para la extracción de datos en el Sistema de Información en Salud para la Atención Básica-2023

Unidad geográfica	Brasil
Período	Enero a diciembre de 2023
Columna en el informe	Indicador 14 = Tipo de producción: Atención individual; Problema/condición evaluada: Atención prenatal, puericultura, puerperio (hasta 42 días), salud sexual y reproductiva, tabaquismo, consumo de alcohol, consumo de otras drogas, salud mental, enfermedades transmisibles: dengue, ITS, lepra, tuberculosis; tamizaje de cáncer de mama y de cuello uterino, tamizaje de riesgo cardiovascular.
	Indicador 15 = tipo de producción: atención individual; problema/condición evaluada: igual que el 14; CIAP/CID: CIAP (50) medicación/prescripción/renovación/inyección, CID (Z760) emisión de recetas y prescripción de repetición.
	Indicador 16 = tipo de producción: procedimiento; procedimiento: prueba rápida de proteinuria, prueba rápida de VIH, prueba rápida de embarazo, prueba rápida de hepatitis C, prueba rápida de sífilis y tamizaje oftalmológico.
	Indicador 17 = tipo de producción: atención individual; problema/condición evaluada: igual que el 14; conducta: agendamiento para el NASF*, agendamiento para grupos, alta del episodio, derivación interna en el día, derivación intersectorial, derivación al CAPS, derivación para hospitalización, derivación al servicio de atención domiciliaria, derivación al servicio especializado, derivación a urgencias, retorno para atención continuada/programada, retorno para consulta programada.
Tipo de cuidado	Consulta programada. prog/cuid. cont., consulta programada, dem. esp. esc. inicial/orient., dem. esp. consulta en el día, dem. esp. Atención de urgencia.
Categoría profesional	Enfermero
Sector de cuidados	UBS, Unidad móvil, Calle, Domicilio, Escuela/guardería, Otros, Polo (academia de salud), Institución/refugio, Unidad penitenciaria o similares, Unidad socioeducativa.
Tipo de equipo de salud	Equipo de Salud Familiar (ESF), Equipo de Agentes Comunitarios de Salud (EACS), NASF*/eMulti, Equipo de Atención Básica (EAB), Equipo de Consultorio en la Calle (ECR), Equipo de Salud Bucal (SB), Equipo AB Penitenciario (EABp), Equipo de Atención Primaria (eAP).

*NASF = Centro de Apoyo a la Salud Familiar

Se reconoce que estos indicadores corresponden a registros administrativos de producción, y no al detallado de la complejidad clínica que implica cada acción. Aun así, representan dimensiones estructurantes del alcance legal y normativo de la práctica de la enfermería en la APS, lo que permite analizar la distribución territorial de estas actividades y su aproximación a las competencias descritas en el referencial internacional. 

### Procedimientos de recolección

La extracción de los datos del SISAB se realizó entre el 3 y el 5 de noviembre de 2024, mediante un proceso automatizado y parametrizado, con la aplicación de un único filtro estandarizado para todos los estados, modalidades de equipo y unidades de atención, de acuerdo con las opciones estructurales disponibles en el propio sistema. Se seleccionaron registros correspondientes a los 12 meses de 2023, limitándose a los profesionales de la categoría de enfermero y abarcando las diferentes modalidades habilitadas en la APS [Equipo de Salud Familiar (ESF), Equipo de Atención Primaria (eAP), Equipo de Consultorio en la Calle (eCR) y otros equipos reconocidos], de acuerdo con los códigos y parámetros estandarizados del Sistema de Gestión de la Tabla de Procedimientos, Medicamentos y OPM (SIGTAP) del Sistema Único de Salud (SUS), tal y como se describe en la Figura 1. Este procedimiento garantizó la uniformidad en la selección y redujo la heterogeneidad derivada de las diferencias en la codificación entre regiones y tipos de equipos.

Tras la extracción, la base de datos se sometió a una doble validación, realizada por un estadístico y por un segundo investigador responsable de la verificación y manipulación de la base, lo que garantizó la integridad, consistencia y aplicabilidad de los registros. Al tratarse de datos agregados por atención, fecha y procedimiento, según los parámetros oficiales del SISAB, no se produjeron duplicidades dentro de los criterios establecidos. Los registros incompletos, inválidos o incompatibles con la APS fueron excluidos automáticamente por el filtro aplicado durante la extracción.

Además, se obtuvieron datos geoespaciales (mapas y coordenadas geográficas) del sitio web del Instituto Brasileño de Geografía y Estadística (IBGE)^19^, utilizando proyección geográfica. Se recopiló información relacionada con el tamaño de la población general de cada unidad de la federación y la población total de Brasil (año censal). Los mapas digitales y sus respectivas coordenadas geodésicas (archivos en formato *.*shp*, *.*shx* e *.*prj*) del territorio nacional y su subdivisión en unidades de la federación (referencia loop 2017) están disponibles en http:downloads.ibge.gov.br/downloads_geociencias.htm. Los archivos con extensión .*shp* adoptan la proyección geográfica SIRGAS_2000, *datum*[“d_SIRGAS_2000”, *spheroid*[“grs_1980”,6378137,298.257222101], *primem*[“*Greenwich*”,0], *unit*[“*degree*”, 0.017453292519943295]].

### Análisis de datos

Esta etapa se dividió en dos fases:

#### Fase 1 - Análisis descriptivo

Se cuantificaron las frecuencias absolutas de las actividades asociadas a los indicadores seleccionados, por unidad de la federación. Esta fase permitió estimar el volumen de acciones realizadas por enfermeros en el ámbito de la APS, clasificadas según los dominios de la EPA (Figura 2). También se utilizó como referencia la fuerza de trabajo de enfermeros vinculados a la APS registrada en el Registro Nacional de Establecimientos de Salud (CNES/Profesionales)^17^. La extracción se realizó con base en los datos disponibles para el mes de julio de 2023, con un total de 100.614 enfermeros en actividad en la APS en el país. 

**Figura 2:** Frecuencia absoluta de enfermeros e intervenciones, en términos de consulta, gestión de episodios complejos de cuidados, autoridad para diagnosticar, prescribir medicamentos, pruebas diagnósticas y tratamientos terapéuticos, en las 26 unidades de la Federación del país y el Distrito Federal. Brasil, 2023

UF	Estado	Número de enfermeros	Indicador 14^*^	Indicador 15^†^	Indicador 16^‡^	Indicador 17^§^
1	Acre	477	304027	4	128971	371863
2	Alagoas	1812	1035600	213	251660	1904659
3	Amapá	556	93576	178	31724	189465
4	Amazonas	1627	1346253	259	452464	2059683
5	Bahia	6604	3873064	1285	583719	6076458
6	Ceará	4582	2963851	1190	450512	5990449
7	Distrito Federal	1618	601603	45	88536	887839
8	Espírito Santo	1861	983349	4666	131563	1586019
9	Goiás	2985	1481209	3664	220240	2538890
10	Maranhão	3686	1940515	650	518498	3427834
11	Mato Grosso	1673	1012717	153	226878	1606304
12	Mato Grosso do Sul	1326	722841	40	178672	1113670
13	Minas Gerais	11927	4975276	3876	771176	9215929
14	Pará	3456	1753622	442	378462	2772583
15	Paraíba	2474	1244622	601	121172	2229152
16	Paraná	6431	3405789	2409	591524	4931203
17	Pernambuco	4148	2983259	165	366765	4587135
18	Piauí	2361	950709	17	48363	1906376
19	Rio de Janeiro	5726	3858600	5765	653215	5390978
20	Rio Grande do Norte	2033	1023808	172	147694	1574712
21	Rio Grande do Sul	6707	3578698	3161	916419	5090050
22	Rondônia	794	372872	46	139102	480213
23	Roraima	381	105395	0	58952	160319
24	Santa Catarina	4842	2541553	1260	735644	3936650
25	São Paulo	18216	7913901	6523	1141077	11242152
26	Sergipe	1236	640694	264	120886	947929
27	Tocantins	1075	421043	164	58906	807368
	Total Geral	100.614	52.128.446	37.212	9.512.794	83.025.882

*Indicador 14 = Consultas; ^†^Indicador 15 = Prescripción de medicaciones; ^‡^Indicador 16 = Prescripciones de pruebas diagnósticas; ^§^Indicador 17 = Derivaciones

#### Fase 2 - Cálculo de indicadores y georreferenciación

A partir de la información totalizada (Figura 2), se elaboraron dos índices:

1) Proporción ajustada: calculada mediante regresión lineal múltiple, con ajuste para el tamaño de la población de enfermeros. Esta técnica se utilizó para obtener el número esperado de acciones de cada uno de los indicadores evaluados, teniendo en cuenta el tamaño de la población de enfermeros (covariada) de cada unidad de la federación, con el objetivo de minimizar el efecto de confusión derivado de la distribución desigual de estos profesionales en el territorio nacional. El índice se calculó de acuerdo con la ecuación:

$$Proporción\;ajustada=\frac{N\;esperadodel\;Indicador\;dela\;Figura\;2}{total\;general\;del\;Indicador}*100$$

La calidad del ajuste de los modelos de regresión lineal se evaluó a partir de R^2^ y R^2^ ajustado (ambos superiores al 90% en los cuatro modelos) y de la homocedasticidad de los residuos (datos no presentados). Este índice permite comparar los indicadores del Figura 2 entre las unidades de la federación.

2) Tasa estandarizada: estimada por estandarización directa^15^, tomando como población estándar el total de la población brasileña (año censal). A partir de los totales presentados en la Figura 2, se calcularon las proporciones brutas [(N observado del indicador/total general del indicador)] y se estimaron los índices de acuerdo con la ecuación:

$$Tasa\;estandarizada=\frac{proporción\;bruta\;del\;indicador}{población\;estándar}*10^{n}\;habitantes/año$$

Este índice permite la comparación en dos direcciones: intra e inter unidades de la federación. Para la representación en mapas temáticos^20^, ese índice fue clasificado en cuartiles, de acuerdo con la distribución. 

Todos los análisis se realizaron con el software Stata^®^, versión 13.2, con aplicación del paquete de comandos *spmap* ^21^ para la etapa de georreferenciación de las tasas estandarizadas. 

### Consideraciones éticas

Al tratarse de un estudio basado exclusivamente en datos secundarios, agregados y de dominio público, no fue necesaria la aprobación de un comité de ética, según la exención prevista en la Resolución CNS nº 510/2016.

## Resultados

La Tabla 1 presenta la distribución proporcional ajustada de las principales acciones realizadas por enfermeros en la APS, considerando el ejercicio de competencias compatibles con la EPA. En todas las unidades federativas, el indicador que más destacó en magnitud fue el 16, es decir, la autoridad para prescribir pruebas diagnósticas y tratamientos terapéuticos, con excepción de los estados de Río de Janeiro y São Paulo.

**Tabla 1:** Proporciones ajustadas de los indicadores, según las unidades de la federación. Brasil, 2023

Unidades Federativas	Proporciones (%) Ajustadas*			
	Indicador 14	Indicador 15	Indicador 16	Indicador 17
Acre	0,7	0,3	1,0	0,8
Amapá	0,8	0,4	1,1	0,8
Amazonas	2,4	2,0	2,6	2,4
Pará	3,0	2,7	3,2	3,0
Rondônia	1,1	0,8	1,4	1,2
Roraima	0,7	0,3	1,0	0,7
Tocantins	1,4	1,0	1,6	1,4
Alagoas	1,8	1,4	2,0	1,8
Bahia	6,6	6,5	6,7	6,6
Ceará	4,5	4,2	4,6	4,5
Maranhão	3,0	2,7	3,1	2,9
Paraíba	2,7	2,3	2,9	2,7
Pernambuco	5,1	4,9	5,3	5,1
Piauí	1,9	1,6	2,2	1,9
Rio Grande do Norte	1,9	1,6	2,2	1,9
Sergipe	1,5	1,2	1,8	1,6
Espírito Santo	2,0	1,7	2,3	2,0
Minas Gerais	8,8	8,7	8,8	8,7
Rio de Janeiro	9,1	9,1	9,1	9,1
São Paulo	24,0	24,7	23,5	24,0
Paraná	5,0	4,8	5,1	5,0
Rio Grande do Sul	4,6	4,4	4,8	4,6
Santa Catarina	3,2	2,9	3,4	3,2
Distrito Federal	3,1	2,8	3,3	3,1
Goiás	3,2	2,9	3,4	3,2
Mato Grosso	2,0	1,7	2,3	2,0
Mato Grosso do Sul	1,6	1,3	1,9	1,7

*Ajuste al tamaño de la población de enfermeros

La Tabla 2 muestra las tasas estandarizadas de los indicadores por unidad de la federación. Las comparaciones interregionales revelan que São Paulo lidera en todos los indicadores (variación entre 5,6 y 8,2), seguido de Minas Gerais, Río de Janeiro y Paraná. 

**Tabla 2:** Distribución de las tasas estandarizadas, según las unidades de la federación. Brasil, 2023

Unidades Federativas	Tasa estandarizada*			
	Indicador 14	Indicador 15	Indicador 16	Indicador 17
Acre	0,3	0	0,6	0,2
Amapá	0,1	0,2	0,2	0,1
Amazonas	1,2	0,3	2,2	1,2
Pará	1,6	0,6	1,9	1,6
Rondônia	0,3	0,1	0,7	0,3
Roraima	0	0	0,3	0,1
Tocantins	0,4	0,2	0,3	0,5
Alagoas	0,9	0,3	1,2	1,1
Bahia	3,5	1,6	2,9	3,4
Ceará	2,7	1,5	2,2	3,4
Maranhão	1,7	0,8	2,6	1,9
Paraíba	1,1	0,7	0,6	1,3
Pernambuco	2,7	0,2	1,8	2,6
Piauí	0,8	0	0,2	1,1
Rio Grande do Norte	0,9	0,2	0,7	0,9
Sergipe	0,6	0,3	0,6	0,5
Espírito Santo	0,9	5,9	0,6	0,9
Minas Gerais	4,5	4,9	3,8	5,2
Rio de Janeiro	3,5	7,3	3,2	3,0
São Paulo	7,1	8,2	5,6	6,4
Paraná	3,1	3,0	2,9	2,8
Rio Grande do Sul	3,2	4,0	4,5	2,9
Santa Catarina	2,3	1,6	3,6	2,2
Distrito Federal	0,5	0,1	0,4	0,5
Goiás	1,3	4,6	1,1	1,4
Mato Grosso	0,9	0,2	1,1	0,9
Mato Grosso do Sul	0,6	0,1	0,9	0,6

*Estandarización por el método directo, población estándar: población total brasileña

La Figura 3 presenta gráficamente la espacialización de las tasas estandarizadas en cuartiles, lo que permite visualizar geográficamente las acciones compatibles con la EPA en la APS. Se observa un patrón de mayor concentración de los indicadores en los estados que componen las regiones Sudeste y Sur, destacando São Paulo, Minas Gerais, Río de Janeiro, Paraná, Rio Grande do Sul y Santa Catarina. Por el contrario, los estados de la región Norte y parte del Nordeste presentan menores concentraciones de los indicadores de rendimiento de la EPA. La visualización evidencia un gradiente geográfico de desigualdad en el ejercicio de la autonomía y de la autoridad clínica de los enfermeros. Este patrón sugiere la existencia de contextos más favorables para la consolidación de la EPA y otros menos favorables, en los que las prácticas clínicas deben ser revisadas y ampliadas, siempre que estén respaldadas por protocolos de regulación municipales e inversiones estructurales en formación especializada y desarrollo profesional continuo. 

**Figura 3:** Distribución geográfica, por unidad federativa, de los indicadores de consulta, gestión de episodios complejos de atención, autoridad para diagnosticar, prescribir medicamentos, pruebas diagnósticas y tratamientos terapéuticos realizados por enfermeros en la Atención Primaria de Salud. Brasil, 2023

## Discusión

Las acciones realizadas por el enfermero en la APS coinciden con las directrices establecidas por el ICN^8^ para la Enfermería de Práctica Avanzada. Este estudio se centró en el análisis de acciones que reflejan la autoridad y la autonomía clínica del enfermero: realización de consultas, derivaciones clínicas, prescripción de pruebas diagnósticas y prescripción de medicamentos en el ámbito de la APS.

La distribución de estos cuatro conjuntos de acciones, en términos de volumen de registros, se produjo en el siguiente orden: consulta de enfermería, derivación de usuarios a otros profesionales y/o servicios de salud, prescripción de pruebas diagnósticas y, por último, prescripción de medicamentos. Al tratarse de un estudio ecológico, las inferencias se refieren a las unidades colectivas y no al desempeño individual de los profesionales. Debe tenerse en cuenta la posibilidad de sesgos relacionados con la calidad del registro y la heterogeneidad estructural de los servicios, aunque se hayan adoptado medidas de estandarización.

La Figura 3 es fundamental para comprender el patrón geográfico de implementación de las cuatro modalidades de acción alineadas con la EPA en Brasil. La imagen muestra la concentración de los cuatro indicadores en el eje Sur-Sudeste, lo que sugiere una mayor institucionalización de las prácticas avanzadas en esos territorios. Por otro lado, los estados de la región Norte y Nordeste, como Acre, Roraima, Piauí y Amapá, se sitúan en los cuartiles inferiores, lo que indica una menor densidad de acciones clínicas compatibles con la EPA y puede reflejar limitaciones estructurales, escasez de protocolos locales o subnotificación. Este gradiente evidencia que, aunque las acciones desarrolladas en Brasil por los enfermeros en la APS están en consonancia con los supuestos de la EPA, persisten importantes desigualdades regionales entre los estados de la federación. Por lo tanto, la expansión de la EPA parece depender de inversiones en el ámbito de la formación profesional, la normalización local y la financiación pública.

El elevado número de consultas de enfermería registradas (52.128.446) demostró la amplia actuación clínica de los enfermeros, especialmente en condiciones que requieren una mayor autonomía diagnóstica, como la salud de la mujer, la salud mental, las enfermedades transmisibles y el tamizaje del cáncer. Esta práctica, aunque ya consolidada en parte del territorio, presentó una distribución desigual entre las unidades de la federación, destacando São Paulo, Minas Gerais, Río de Janeiro, Paraná, Bahía y Río Grande do Sul, como se identifica en la Figura 3 (cuartil superior del análisis espacial). Estos hallazgos refuerzan la existencia de contextos más favorables para la consolidación de la EPA en estos estados.

Estudios nacionales corroboran este escenario al destacar la percepción positiva de la autonomía del enfermero en la APS^14^ ^),(^ ^22^ ^)-(^ ^24^, y evidenciar que, cuando están respaldados por normativas y evidencia científica, estos profesionales asumen un papel protagonista en la atención clínica, sobre todo en áreas sensibles como las infecciones de transmisión sexual (ITS) y la atención a la salud de la mujer^25^. 

Por lo tanto, los resultados presentados en este estudio no corresponden a un modelo formalmente reconocido de Enfermería de Práctica Avanzada en Brasil, dado que esta función carece de una regulación específica, pero evidencian dimensiones del ejercicio clínico del enfermero que se alinean con las competencias descritas en referencias internacionales y apuntan a un potencial de desarrollo. Estas acciones, realizadas por enfermeros generalistas en el ámbito del alcance ampliado previsto en la legislación brasileña, revelan patrones de madurez clínica y protagonismo que pueden subsidiar la construcción de marcos formativos y regulatorios alineados con las directrices internacionales de la EPA. Los resultados demuestran un conjunto significativo de prácticas que dialogan con dominios avanzados, lo que ofrece una base empírica para orientar debates y propuestas futuras.

Además, se identificaron 83.025.882 registros de derivaciones, lo que evidencia el papel del enfermero como coordinador de la atención y articulador de los flujos asistenciales. El análisis por cuartiles mostró una mayor concentración de estos registros en los estados de Ceará, Bahía, Minas Gerais, São Paulo, Río de Janeiro y Río Grande do Sul, como se muestra en la Figura 3. Esta concentración puede estar relacionada con una mayor institucionalización de los protocolos clínicos municipales que respaldan las decisiones clínicas de los enfermeros. La literatura^26^ corrobora este hallazgo al destacar el papel del “enfermero de enlace” como elemento clave en la articulación efectiva de las redes de atención en salud, especialmente en los procesos de derivación y contrarreferencia.

En lo que respecta a la prescripción de pruebas diagnósticas y tratamientos terapéuticos (n=9.512.794), los resultados indicaron una mayor expresividad en los estados de Minas Gerais, Río de Janeiro, São Paulo, Paraná, Santa Catarina y Río Grande do Sul, lo que sugiere mejores condiciones estructurales y organizativas, como una mayor cobertura de la Estrategia de Salud Familiar (ESF) y capacitación técnica. 

Por otro lado, los resultados relativos a la prescripción de medicamentos por parte de enfermeros (n=37.212) fueron relativamente bajos en comparación con el volumen de consultas y derivaciones. Aunque la PNAB autoriza la prescripción por enfermeros, la aplicación efectiva de esta competencia depende de los protocolos locales, lo que genera disparidades entre los estados. La Figura 3 muestra una mayor incidencia de recetas en los estados de São Paulo, Río de Janeiro, Espírito Santo, Minas Gerais, Goiás y Paraná. Estudios anteriores^9^ ^),(^ ^14^ ^),(^ ^27^ ^)^ indican que los enfermeros se sienten preparados y reconocen la prescripción como un avance en la autonomía profesional, siempre que esté respaldada por normativas técnicas. Por lo tanto, las bajas cifras nacionales relativas a la prescripción por parte de enfermeros pueden estar relacionadas con la inexistencia o la desactualización de los protocolos municipales, la inseguridad jurídica y las barreras institucionales. Cabe destacar que la prescripción de medicamentos, en el contexto de la EPA en la APS, representa un avance en consonancia con los principios de integralidad y resolución de la atención^9^ ^),(^ ^14^. 

En este contexto, el ICN recomienda incluir la prescripción de medicamentos en el ámbito de actuación de la EPA, a nivel mundial, siempre que vaya acompañada de formación y regulación específicas, así como de apoyo institucional^8^. 

En Europa, la implementación de la EPA presenta una imagen heterogénea, ya que solo 11 de los 35 países de la región paneuropea cuentan con legislación formal sobre el tema^28^. Todavía existe una gran variabilidad en la definición de EPA, especialmente en lo que se refiere a la nomenclatura, el ámbito de actuación, la regulación, los requisitos educativos para el ejercicio de la función y las estrategias de financiación^28^, lo que compromete su realización. En países como Francia, por ejemplo, la ambigüedad de las funciones, unida a la indefinición del modelo de financiación y del apoyo institucional, condiciona el desarrollo de la EPA, una situación que también se da en países como Inglaterra, donde la práctica está ampliamente establecida^29^ ^)-(^ ^30^. En los países nórdicos y de Oceanía, como Australia, Finlandia y Noruega, existe una mayor estandarización en la formación, regulación y reconocimiento de la EPA como estratégica para los sistemas de salud^31^. Un estudio europeo destaca la importancia de combatir el aislamiento profesional mediante redes de contacto y tutorías, como estrategias esenciales para promover la sostenibilidad de la fuerza laboral de enfermería de práctica avanzada^32^. En la región de las Américas, el 64% de los países han implementado funciones de enfermería avanzada^33^, Sin embargo, los países de América Latina deben acentuar la inversión en esta estrategia^14^ ^),(^ ^34^, valorizando el diálogo entre las partes interesadas^35^.

En los contextos en los que el debate sobre la EPA aún es incipiente, aunque se considera una iniciativa prometedora^36^ ^)-(^ ^37^, es esencial fortalecer la base educativa, política e institucional de la enfermería para garantizar la introducción sostenible y eficaz de la práctica avanzada^38^. Además, la formación continua se consideró un factor clave para fomentar la confianza en el desempeño de la función de enfermería de práctica avanzada, tal y como se demostró en estudios centrados en el análisis del tipo de intervención educativa a lo largo del tiempo de implementación^39^. En el caso brasileño, es precisamente la base educativa la que debe convertirse en el centro de las acciones estratégicas de los líderes de enfermería, ya que, aunque se observa un número creciente de intervenciones clínicas avanzadas en la APS, la ausencia de normativas nacionales específicas, sumada a la dependencia de la regulación local, compromete la equidad en el ejercicio de la EPA entre los estados. El estudio también señaló que existe una masa crítica instalada para avanzar: con más de 100.000 enfermeros trabajando en la APS, a lo que debe asociarse un sólido sistema de formación en enfermería (cursos de grado, másteres y doctorados académicos y profesionales)^40^ y líderes organizados en especialidades y sociedades científicas. 

A pesar del alcance nacional y la solidez de los datos utilizados, este estudio presenta algunas limitaciones que deben tenerse en cuenta. La principal de ellas se refiere a la posible subnotificación de las acciones registradas en los sistemas oficiales de información sanitaria, especialmente en las regiones Norte y Nordeste, como se observa en los estados de Piauí, Roraima y Acre, donde la magnitud de los indicadores estandarizados fue igual a cero o extremadamente baja. Este escenario puede no reflejar la inexistencia de prácticas clínicas compatibles con la EPA, sino más bien debilidades en el registro, inconsistencias en los sistemas de información o la ausencia de codificación estandarizada en las unidades de salud. Además, la naturaleza ecológica y agregada del análisis impide inferir causas o interpretaciones individualizadas. Por lo tanto, se requieren estudios adicionales que proporcionen información desagregada.

Cabe destacar también que las acciones analizadas no son, por sí mismas, equivalentes a la EPA. En el contexto brasileño, muchas de ellas forman parte del ámbito ampliado del enfermero generalista en el SUS, tal y como se establece en la PNAB y en la ley del ejercicio profesional, y no implican necesariamente competencias avanzadas tal y como las define el ICN. 

Aun así, la ejecución consistente y generalizada de las acciones analizadas en este estudio pone de manifiesto la madurez clínica que los enfermeros han ido desarrollando en la APS, lo que demuestra capacidad técnica, responsabilidad y protagonismo en la atención. Estos hallazgos revelan, además, un potencial significativo para orientar las políticas de formación, regulación y ampliación del ámbito profesional, al ofrecer datos concretos sobre la actuación real de la enfermería en el territorio nacional. Así, los indicadores analizados fundamentan claramente el papel central de los enfermeros en la APS brasileña y contribuyen a los debates estratégicos sobre la regulación y el fortalecimiento de la práctica avanzada en el país, sin caracterizar directamente tales acciones como Enfermería de Práctica Avanzada, pero evidenciando dimensiones clínicas que dialogan con este referencial internacional.

## Conclusión

Este estudio ecológico ofrece una visión nacional de la distribución y la intensidad de las acciones clínicas realizadas por enfermeros en la atención primaria de salud. Aunque no permite inferir la complejidad del razonamiento clínico ni caracterizar las competencias avanzadas en los términos definidos por el ICN, los patrones observados revelan un campo de práctica sólido, en el que la enfermería desempeña funciones centrales en la atención y acumula experiencia que puede sustentar avances estructurales.

La heterogeneidad territorial identificada refleja la influencia de las capacidades organizativas, la infraestructura y la disponibilidad de profesionales, lo que evidencia áreas que requieren fortalecimiento y cualificación. Además, la presencia de más de 100.000 enfermeros que trabajan en la APS, junto con una sólida estructura formativa con una masa crítica consolidada en los niveles de posgrado académico y profesional, configura un escenario estratégico para el desarrollo de programas de formación avanzada, incluidos másteres profesionales orientados a la práctica clínica ampliada, alineados con las necesidades del SUS.

Los hallazgos apuntan a vías concretas para mejorar el ejercicio clínico de la enfermería en el país, incluyendo la cualificación de los sistemas de registro, la estandarización de la codificación, la armonización de los protocolos y la realización de estudios cualitativos y de métodos mixtos que profundicen en la comprensión de los procesos de toma de decisiones y las condiciones que configuran la autonomía profesional en la APS. Al ofrecer evidencia empírica sobre el trabajo clínico de los enfermeros a escala nacional, este estudio contribuye a los debates estratégicos sobre el fortalecimiento y la ampliación del alcance clínico, reforzando la relevancia de las medidas que avanzan hacia la regulación y la valorización de la autonomía y la autoridad clínica de la enfermería, elementos centrales para la eficacia y la sostenibilidad de la atención primaria en Brasil.

## Data Availability

Todos los datos generados o analizados durante este estudio están incluidos en este artículo publicado.
